# Aflatoxin M_1_ in Milk and Aflatoxin B_1_ in Animal Feed in Bangladesh: A Systematic Review, Meta‐Analysis and Literature‐Based Dietary Risk Assessment

**DOI:** 10.1002/vms3.71284

**Published:** 2026-10-16

**Authors:** MD. Al Fattah, Md. Ahsan Habib

**Affiliations:** ^1^ Department of Animal Science and Nutrition Faculty of Veterinary and Animal Science Hajee Mohammad Danesh Science and Technology University Dinajpur Bangladesh

**Keywords:** aflatoxin B_1_, aflatoxin M_1_, animal feed, meta‐analysis, milk, mycotoxins, risk assessment, uncertainty analysis

## Abstract

**Background:**

Aflatoxin M_1_ (AFM_1_), the carcinogenic metabolite of aflatoxin B_1_ (AFB_1_), passes into the milk of animals fed mould‐contaminated feed. Bangladeshi feed and milk surveillance have developed separately, and integrated dietary‐risk estimates are scarce.

**Objective and design:**

To pool AFM_1_ occurrence in Bangladeshi milk, summarise feed AFB_1_ and characterise age‐stratified dietary risk. The assessment is literature‐based and modular: no Bangladeshi study reports paired feed and milk data, so compartments are joined by parameter transfer.

**Methods:**

Prevalences were pooled by DerSimonian–Laird random‐effects models with prediction intervals, subgroup, meta‐regression, leave‐one‐out and small‐study analyses. Exposure was modelled deterministically and probabilistically (100,000 iterations), with uncertainty tabulated following EFSA guidance.

**Results:**

Across 20 datasets (*N* = 605), pooled prevalence was 58.7% (95% CI 51.6%–65.5%; *I* ^2^ = 61.9%; 95% prediction interval 32.1%–81.1%). Heterogeneity was structured by region (*p* = 0.001), not milk type (*p* = 0.980). The sample‐weighted mean was 143 ng L^−1^ (geometric mean 92); 83% of milk exceeded the EU maximum level. Reconstruction from feed AFB_1_ predicted 24–282 ng L^−1^, containing 18 of 20 dataset means. Margins of exposure were 95 (infants) and 175 (toddlers), with probabilistic medians 157 and 286; the deterministic hazard index exceeded unity in every age group, the probabilistic median in every group except adults.

**Conclusions:**

Infant and child margins of exposure remain far below the 10,000 benchmark under every scenario and robustness check. The estimate is screening‐level, resting on transferred parameters and ELISA‐based measurement. Monsoon‐aware feed management, enforcement of the national milk standard and paired feed–milk surveillance are indicated.

## Graphical abstract

A systematic review and meta‐analysis of 20 datasets (*N* = 605) revealed 58.7% AFM_1_ prevalence in Bangladeshi milk, with a mean concentration of 143 ng/L. Feed AFB_1_‐based reconstruction indicated potential milk contamination. Dietary risk assessment identified critically low margins of exposure, particularly among infants and children. Strengthened feed management, milk‐standard enforcement, and paired feedmilk surveillance are urgently needed.

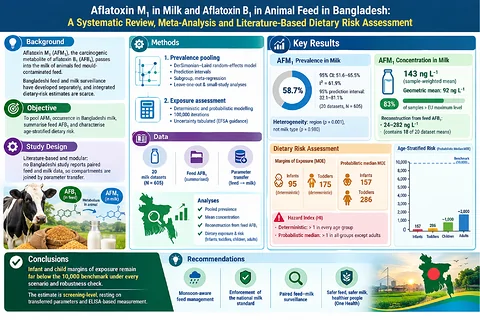

**Abbreviations**
AFB_1_
: aflatoxin B_1_
AFM_1_
: aflatoxin M_1_
BFSA: Bangladesh Food Safety Authority
BMDL_10_
: benchmark dose lower confidence limit (10%)
BSTI: Bangladesh Standards and Testing Institution
CI: confidence interval
EDI: estimated daily intake
ELISA: enzyme‐linked immunosorbent assay
HBsAg: hepatitis‐B surface antigen
HCC: hepatocellular carcinoma
HI: hazard index
ML: maximum level
MOE: margin of exposure
UHT: ultra‐high temperature

## 1 Introduction

Aflatoxins are difuranocoumarin secondary metabolites produced principally by *Aspergillus flavus* and *Aspergillus parasiticus*. Aflatoxin B_1_ (AFB_1_) is the most prevalent congener in food and feed and the most potent naturally occurring hepatocarcinogen, classified by the International Agency for Research on Cancer as a Group 1 human carcinogen (IARC 2012; Marchese et al. 2018). When a lactating animal ingests AFB_1_‐contaminated feed, hepatic cytochrome‐P450 hydroxylation converts part of the toxin to aflatoxin M_1_ (AFM_1_), which is secreted into milk. AFM_1_ retains about one‐tenth of the carcinogenic potency of the parent compound, but it resists pasteurisation and ultra‐high‐temperature (UHT) processing and therefore reaches the consumer intact (Iqbal et al. 2015; Becker‐Algeri et al. 2016). Milk and dairy products are consumed across all age strata and, per unit body weight, disproportionately by infants and young children whose detoxification capacity is still developing. AFM_1_ is accordingly a food‐safety hazard of particular public‐health importance, amplified where chronic hepatitis‐B infection acts multiplicatively with aflatoxin to raise hepatocellular‐carcinoma (HCC) risk (Liu and Wu 2010; EFSA Panel on Contaminants in the Food Chain (CONTAM) 2020). Whether that hazard becomes a population burden depends on three further conditions: the agro‐climatic environment governing fungal growth, the structure of the dairy supply chain, and the regulatory framework in force. In Bangladesh, all three align unfavourably.

Bangladesh combines the agro‐climatic, structural and dietary determinants of aflatoxin exposure to an unusual degree. Its tropical monsoon climate sustains high temperature and humidity through grain filling, harvest and on‐farm storage, favouring *Aspergillus* proliferation and toxin accumulation (Dawlatana et al. 2002; Roy et al. 2022). Dairy and poultry production are expanding but remain dominated by smallholder and informal supply chains with limited cold‐chain infrastructure, fragmented quality control and weak enforcement. Maize, the cereal most susceptible to aflatoxigenic fungi, is now the principal energy ingredient in compound feeds, placing a high‐risk commodity at the base of the dairy value chain (Roy et al. 2022). Aflatoxins also contaminate rice, the national staple, extending exposure beyond the dairy chain (Ali 2019). Consistent with these conditions, Bangladeshi surveys have repeatedly found AFB_1_ in poultry feed, beef‐cattle rations and feed ingredients at concentrations that breach international feed limits (Lubna et al. 2018; Kamal et al. 2022; Phillips et al. 1996), and urinary biomonitoring of adults, mill workers and pregnant women confirms widespread dietary exposure to aflatoxins (Ali et al. 2016, 2023).

The regulatory benchmarks against which Bangladeshi milk must be judged have themselves been consolidated and, for the most vulnerable consumers, tightened. In the European Union, Commission Regulation (EU) 2023/915 replaced Regulation (EC) No 1881/2006; it retains a maximum level (ML) of 0.050 µg kg^−1^ for AFM_1_ in raw milk, heat‐treated milk and milk for the manufacture of milk‐based products, and sets half that value, 0.025 µg kg^−1^, for infant formula, follow‐on formula and foods for special medical purposes intended for infants (European Commission 2023; Mousavi Khaneghah 2023). The Codex general standard for contaminants (CXS 193‐1995) and the United States Food and Drug Administration both apply 0.5 µg kg^−1^, a 10‐fold more permissive figure, and the Bangladesh Standards and Testing Institution (BSTI) and Bangladesh Food Safety Authority (BFSA) limits align with the Codex value (European Commission 2006; FAO/WHO Joint Expert Committee on Food Additives (JECFA) 2018; Bangladesh Food Safety Authority 2017; Codex Alimentarius Commission 2019). Intermediate national values exist; the Iranian National Standards Organization, for example, applies 100 ng L^−1^ (Rahimzadeh Barzoki et al. 2023). For feed, Directive 2002/32/EC permits 20 µg kg^−1^ AFB_1_ in feed materials but only 5 µg kg^−1^ in complementary and complete feed for dairy animals, an asymmetry that reflects the carry‐over risk specific to lactating cattle. The same Bangladeshi milk sample may therefore comply with the national limit while lying an order of magnitude above the level the European Union accepts for an infant, so the choice of benchmark materially determines what counts as a violation.

The international occurrence literature supplies the comparative frame for Bangladeshi values and is considerably better developed. Seasonally resolved surveys of retail cow milk in Gorgan, Iran, detected AFM_1_ in 72.2% of 180 samples at 3.5–357 ng L^−1^ (Rahimzadeh Barzoki et al. 2023), and comparable prevalences are reported for processed dairy in the same country: 84% of UHT and 87.3% of pasteurised milk in Hamadan province (Mozaffari Nejad et al. 2019), 86% of yoghurt samples, of which 18% exceeded 50 ng kg^−1^ (Heshmati et al. 2020), and cheese, in which concentration during curd formation raises the residue above that of the source milk (Mozaffari Nejad et al. 2020). Two features of this work bear on the present study. First, prevalence remains high in heat‐treated products, confirming that processing offers the consumer no protection. Second, these studies characterise risk through estimated daily intake (EDI), hazard index (HI) and margin of exposure (MOE) rather than through prevalence alone, a framework also applied to aflatoxins in other staples such as wheat flour (Heshmati et al. 2023). The present assessment adopts the same convention, so that Bangladeshi estimates can be read alongside published values rather than in isolation. What the Bangladeshi literature lacks is not occurrence data but this translation into exposure.

Bangladeshi studies have detected AFM_1_ in 43%–79% of milk samples, with raw‐milk means ranging from a few nanograms per litre in some divisions to almost 700 ng L^−1^ in the most affected Dhaka samples (Sumon et al. 2021; Rahman et al. 2020; Tarannum et al. 2022; Ali et al. 2023), an order of magnitude above the EU ML of 50 ng L^−1^ and, at the extreme, above the more permissive Codex and BSTI/BFSA limit of 500 ng L^−1^ (European Commission 2006; FAO/WHO Joint Expert Committee on Food Additives (JECFA) 2018; Bangladesh Food Safety Authority 2017). This evidence is fragmented in three consequential ways. First, the feed and milk literatures have developed in isolation, so the upstream contamination that drives milk residues has never been measured alongside the AFM_1_ it produces in the same animals. Second, prevalence estimates vary widely across regions, milk types and analytical methods, and no random‐effects synthesis has reconciled them into a national figure with a stated uncertainty. Third, dietary‐risk characterisations using Bangladesh‐specific milk‐intake and body‐weight parameters, rather than values imported from Western dairy economies, are largely absent, leaving the carcinogenic burden borne by the most vulnerable consumers unquantified.

This study addresses those gaps through a systematic review, meta‐analysis and literature‐based dietary risk assessment of the feed‐to‐human pathway. The nature of the exercise should be stated at the outset. The assessment is modular: each compartment is populated from a separate body of published evidence and joined to the next by parameter transfer rather than by paired measurement (Figure 1; Section 2.1). No Bangladeshi study reports AFB_1_ in feed and AFM_1_ in milk from the same herds, farms or sampling frame, so the work cannot establish an empirical feed‐to‐milk association within Bangladeshi production systems. What it can do is estimate each compartment as reliably as the published evidence allows, test whether the compartments are quantitatively consistent under an explicit transfer model, and propagate the resulting uncertainty into the human‐health estimates.

**FIGURE 1:**
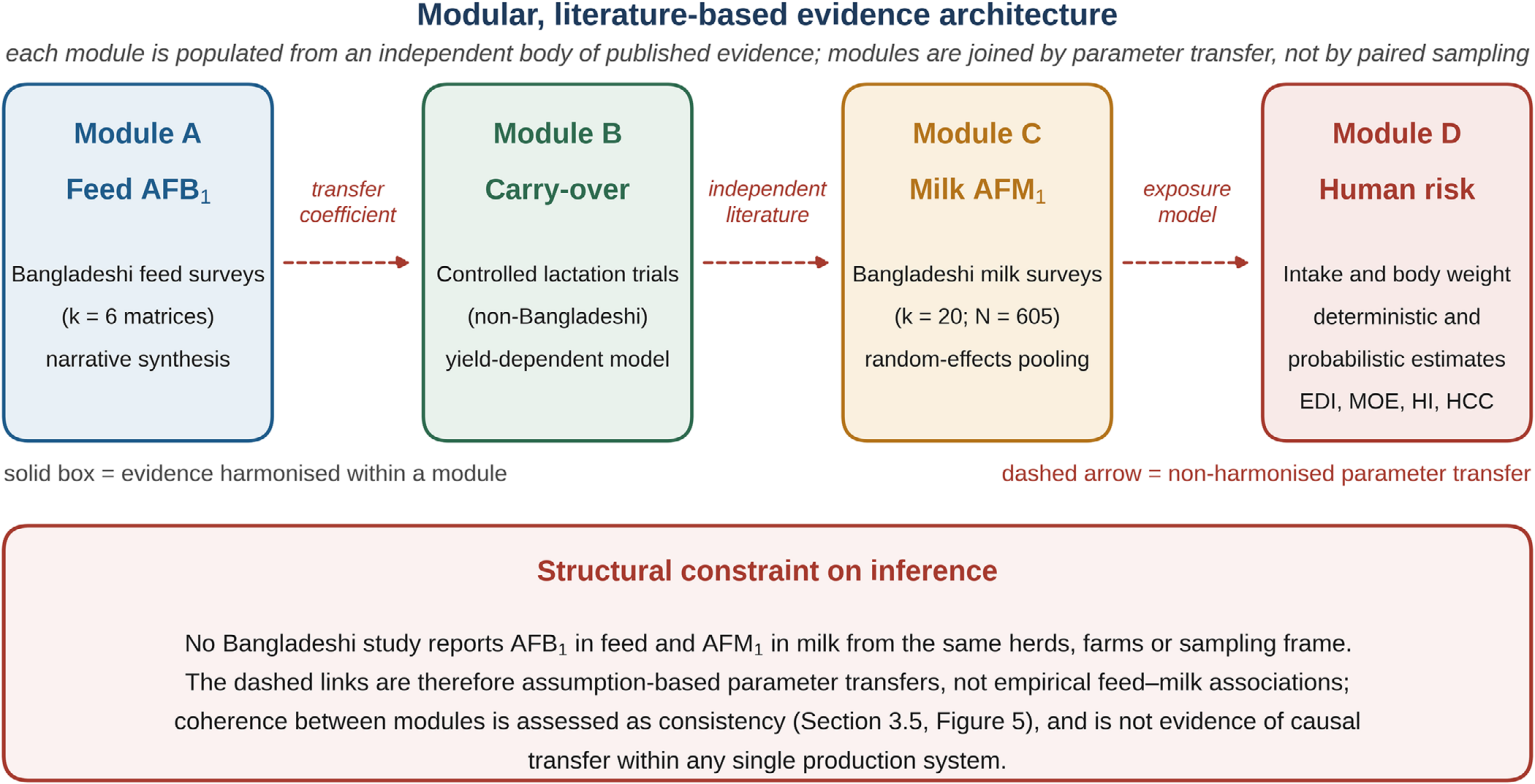
Modular, literature‐based evidence architecture of the assessment. Each module is populated from an independent body of published evidence and joined to the next by parameter transfer (dashed arrows), not by paired sampling. No Bangladeshi study reports AFB_1_ in feed and AFM_1_ in milk from the same herds or sampling frame; the feed and milk modules are therefore assessed for quantitative consistency (Section 3.5) rather than linked causally, and human exposure is driven by the milk module alone.

The specific objectives are to (i) pool the national prevalence of AFM_1_ in Bangladeshi milk under a DerSimonian–Laird random‐effects model, reporting prediction intervals alongside confidence intervals (CIs) and quantifying and explaining heterogeneity (DerSimonian and Laird 1986); (ii) summarise AFB_1_ and co‐occurring mycotoxins across Bangladeshi feed types and describe their monsoon seasonality; (iii) test whether the observed milk AFM_1_ distribution is quantitatively reconcilable with the reported feed AFB_1_ burden under published, yield‐dependent carry‐over coefficients (Britzi et al. 2013), treating the result as a consistency check rather than a causal linkage; and (iv) translate the pooled residue levels into age‐stratified deterministic and probabilistic estimates of daily intake, MOE, HI and incremental HCC risk, using Bangladesh‐specific exposure parameters and an HBsAg (hepatitis‐B surface antigen)‐weighted potency factor, with a structured analysis of the uncertainty attaching to each estimate. The result is a screening‐level national evidence base for risk‐based control of aflatoxins along the Bangladesh dairy chain.

## 2 Materials and Methods

### 2.1 Study Design and Evidence Architecture

This is a literature‐based, modular risk assessment (Figure 1). Four modules were populated independently. Module A covers AFB_1_ and co‐occurring mycotoxins in Bangladeshi feeds and feed ingredients, and is synthesised narratively because the primary reports are matrix‐heterogeneous and rarely give the sample‐level detail required for pooling. Module B is the feed‐to‐milk carry‐over coefficient, taken from controlled lactation trials conducted outside Bangladesh. Module C is AFM_1_ occurrence in Bangladeshi milk, synthesised quantitatively by random‐effects meta‐analysis. Module D is human dietary exposure and risk, modelled from Module C together with Bangladesh‐specific consumption and body‐weight parameters.

Three features of this architecture constrain inference and are carried explicitly through the Sections 3 and 4. First, the modules are not harmonised: they draw on different studies, sampling frames, years, regions and laboratories, and no dataset contributes to more than one module. Second, the links between modules (dashed arrows in Figure 1) are parameter transfers, not measured associations; the carry‐over coefficient of Module B derives from high‐yielding cows under controlled feeding and is applied to Bangladeshi smallholder conditions on the assumption that the underlying hepatic biotransformation is conserved. Third, human exposure is driven by Module C alone: the feed data do not enter the exposure calculation and serve only to characterise the upstream hazard and to test consistency (Section 2.4).

### 2.2 Data Sources, Eligibility and Study Appraisal

Following PRISMA 2020 (Page et al. 2021), PubMed, Scopus, Web of Science and ScienceDirect were searched for the period January 2000 to January 2026, combining (‘aflatoxin M1’ OR AFM1 OR ‘aflatoxin B1’ OR mycotoxin) AND (milk OR dairy OR feed) AND Bangladesh. Records were eligible if they reported the prevalence or concentration of AFM_1_ in Bangladeshi milk, or of AFB_1_ or other mycotoxins in Bangladeshi feed, with an extractable sample size. Human breast‐milk studies were excluded from the milk meta‐analysis as a different exposure pathway but are cited as biomonitoring evidence. The selection process is summarised in Figure 2.

**FIGURE 2:**
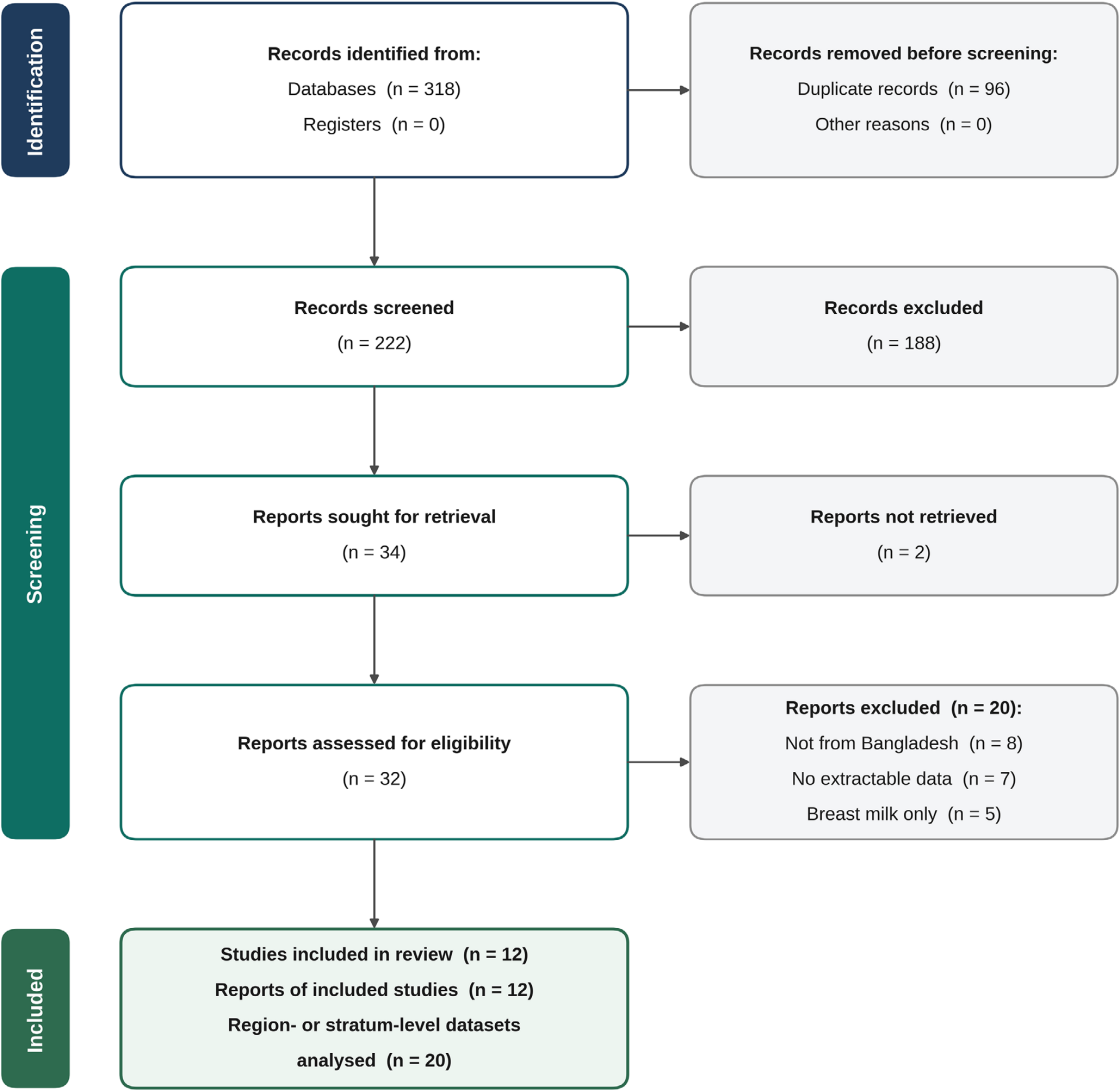
PRISMA 2020 flow diagram of the identification, screening and inclusion of studies reporting aflatoxin M_1_ in Bangladeshi milk. Eligible reports provided the 20 region‐ or stratum‐level datasets analysed.

The searches were run on 31 January 2026 with no language or document‐type restriction. No trial or systematic‐review registers were searched and the review was not prospectively registered (Figure 2, registers *n* = 0). The full database‐specific Boolean strings, including the two complementary ScienceDirect runs required by that platform's truncation and eight‐connector limits, are given in Table S6 together with the field tags, filters and number of records returned by each database. The reference lists of all reports assessed at full text, and of relevant reviews, were hand‐searched and yielded no further eligible studies, so the identification arm of Figure 2 comprises database records only. The completed PRISMA 2020 checklist is provided as Table S5.

Records were exported in RIS format and deduplicated automatically on the digital object identifier and on the combination of first author, year, journal, volume and start page, followed by manual inspection of all flagged near‐matches and of every title returned by more than one database. Of the 318 records identified, 96 were removed as duplicates, leaving 222 unique records (Figure 2). Conference abstracts subsequently published in full were treated as duplicates of the full report.

Titles and abstracts were screened independently and in duplicate by two reviewers (M.A.F. and M.A.H.) using a piloted form, and 188 records were excluded. The full texts of the 34 records sought for retrieval were assessed independently and in duplicate by the same two reviewers; two could not be obtained, leaving 32 assessed for eligibility. Disagreements were resolved by discussion against the written eligibility criteria. Because the review team comprised only these two authors, no third adjudicator was available, and a conservative rule was pre‐specified: any unresolved record was carried forward to full‐text assessment rather than excluded on a single reviewer's judgement. All decisions and reasons for exclusion were logged. Twenty reports were excluded at full text (not conducted in Bangladesh, *n* = 8; no extractable sample size or prevalence, *n* = 7; human breast milk only, *n* = 5), leaving 12 eligible reports.

Data were extracted by one reviewer and checked in full against the source report by the second. The eligible reports were then screened for duplicate publication and sample overlap: where two reports described the same sampling frame, division, collection period and analytical laboratory, the more completely reported was retained, so that no milk sample contributes more than once to the pooled estimate.

Where a report presented separable strata by division, milk type or collection site, each stratum was entered as a distinct dataset; the 20 analysed datasets therefore derive from a smaller number of independent reports, and this clustering is addressed in Sections 2.7 and 3.8. Each dataset was appraised with a prevalence‐adapted Newcastle–Ottawa instrument (Wells et al. 2021) covering representativeness of the sampling frame, adequacy of sample size, description and validation of the analytical method, reporting of limits of detection and recovery, and completeness of outcome reporting; the domain definitions and the resulting classification of every dataset are given in Table S1. Datasets were classified as at low, moderate or high risk of bias (Table 1), and a pre‐specified sensitivity analysis restricted the synthesis to datasets originating from fully referenced, peer‐reviewed reports.

**TABLE 1:** Characteristics and risk‐of‐bias classification of the 20 Bangladeshi AFM_1_‐in‐milk datasets included in the meta‐analysis.

ID	Division/area	Source	Milk type	*n*	Prev. (%)	Mean (ng L^−1^)	Risk of bias
1	Sylhet	Sumon et al. (2021)	Raw	26	72.0	53.7	Low
2	Dhaka	Sumon et al. (2021)	Raw	28	71.0	37.8	Low
3	Rajshahi	Sumon et al. (2021)	Raw	26	74.0	34.8	Low
4	Chattogram	Sumon et al. (2021)	Raw	25	45.0	6.6	Low
5	National	Sumon et al. (2021)	Pasteurised	15	100.0	106.0	Low
6	National	Sumon et al. (2021)	UHT	15	100.0	73.0	Low
7	Dhaka	Tarannum et al. (2022)	Raw	50	70.0	699.1	Low
8	Dhaka	Tarannum et al. (2022)	Pasteurised	25	52.0	99.8	Low
9	Dhaka	Tarannum et al. (2022)	UHT	25	20.0	35.5	Low
10	Dhaka	Rahman et al. (2020)	Raw	50	64.0	152.0	Moderate
11	Dhaka	Rahman et al. (2020)	Pasteurised	25	48.0	88.0	Moderate
12	Chattogram (Bakalia)	Ali et al. (2023)	Raw	30	47.0	190.0	Moderate
13	Chattogram (Khulshi)	Ali et al. (2023)	Raw	30	40.0	108.4	Moderate
14	Chattogram (Pahartali)	Ali et al. (2023)	Raw	30	43.0	189.3	Moderate
15	Pabna–Sirajganj	Milk‐shed survey	Raw	45	75.0	120.0	High
16	Mymensingh	Dairy‐belt survey	Raw	40	68.0	88.0	High
17	Bogura	Regional survey	Raw	35	66.0	95.0	High
18	Khulna	Regional survey	Raw	30	58.0	64.0	High
19	Barishal	Regional survey	Raw	25	50.0	58.0	High
20	Rangpur	Regional survey	Raw	30	55.0	70.0	High
	Total			605			

*Note*: All datasets used competitive ELISA; limits of detection, recovery and the completed appraisal instrument are given in Table S1. Risk of bias: Low = fully referenced peer‐reviewed report with described sampling frame and method; Moderate = peer‐reviewed report with incomplete methodological reporting; High = survey not attached to a fully referenced peer‐reviewed report. Datasets 1–9 derive from two reports and datasets 10–14 from two further reports; datasets are therefore clustered rather than independent.

### 2.3 Meta‐Analytic Model

Study‐level prevalences *p* were logit‐transformed (Equation 1) with variance *v* = 1/[*n p*(1 − *p*)]; datasets reporting 100% positivity received a 0.5 continuity correction. Pooling used the DerSimonian–Laird random‐effects estimator, with *τ* ^2^ from Cochran's *Q* and inconsistency summarised by *I* ^2^ (Equation 2); pooled logits were back‐transformed (Equation 3). Because *I* ^2^ describes the proportion of variability attributable to between‐dataset differences rather than the spread of true prevalences, a 95% prediction interval was also computed (Equation 4) and is reported alongside every pooled estimate based on three or more datasets (Higgins et al. 2009). The logit scale was preferred to the arithmetic scale because it stabilises the variance of proportions near the boundaries and returns back‐transformed limits within [0,1] (Barendregt et al. 2013).

Pre‐specified subgroup analyses examined milk type (raw, pasteurised, UHT), region (Dhaka, Chattogram, other divisions and national) and risk‐of‐bias class, with between‐subgroup differences tested by *Q*‐between. Random‐effects meta‐regression (weights 1/(*v* + *τ* ^2^)) examined mean concentration, dataset size and milk type as continuous or binary moderators; residual *I* ^2^ and the proportion of Q explained are reported. Given the number of datasets, meta‐regression is reported as exploratory, and no moderator model with more than one covariate was fitted (Higgins et al. 2009).

*Unit of analysis*: The unit of analysis was the reported dataset, defined as a division‐, site‐ or milk‐type‐specific stratum for which both a prevalence and a denominator could be extracted. As Table 1 records, the 20 datasets derive from 10 distinguishable sources: four peer‐reviewed reports contributing six, three, two and three strata respectively, and six regional surveys contributing one stratum each. Strata drawn from a common report are not statistically independent, since they may share a sampling frame, laboratory, enzyme‐linked immunosorbent assay (ELISA) kit, operator and season. The inverse‐variance weights of Equation 3 assume independence and therefore credit the evidence base with more information than it contains, making the nominal CI too narrow. Two unit‐of‐analysis sensitivity analyses were specified to bound this effect: aggregation, in which positives and denominators were summed within each source to give one record per source before pooling; and selection, in which only the largest stratum from each source was retained. Both are reported in Section 3.8.

Three subgroup comparisons were specified in advance (region, milk type and risk‐of‐bias class), and each was tested formally by *Q*‐between on the logit scale using subgroup‐specific *τ* ^2^; no further subgroup was examined post hoc. Because three tests were applied to the same pooled dataset, Holm‐adjusted *p* values are reported alongside the nominal values, and subgroup results are interpreted as hypothesis‐generating rather than confirmatory, consistent with the exploratory status of the meta‐regression.

(1) $$y_{i}=\ln\left(\frac{p_{i}}{1-p_{i}}\right),\;v_{i}=\frac{1}{n_{i}p_{i}\left(1-p_{i}\right)}$$

(2) $$Q=\Sigma w_{i}{\left(y_{i}-\bar{y}\right)}^{2},\tau^{2}=\max0,(Q-\text{df})/C,I^{2}=\left(Q-\text{df}\right)/Q\times 100\%$$

(3) $${\hat{p}}_{\mathrm{pooled}}=\text{expit}\left(\Sigma w_{i}^{*}y_{i}/\Sigma w_{i}^{*}\right),w_{i}^{*}=1/\left(v_{i}+\tau^{2}\right)$$

(4) $$95\%\text{PI}=\text{expit}({\hat{y}}_{\mathrm{pooled}}\pm t_{\left(df-1,0.975\right)}\sqrt{(}\tau^{2}+{\text{SE}}^{2}\left({\hat{y}}_{\mathrm{pooled}}\right)))$$

### 2.4 Feed‐to‐Milk Carry‐Over and the Mechanistic Consistency Check

Carry‐over (the percentage of ingested AFB_1_ recovered as AFM_1_ in milk) was taken from controlled trials (Equation 5), with its milk‐yield dependence modelled exponentially (Equation 6) to accommodate the low daily yields typical of Bangladeshi smallholder cattle (Britzi et al. 2013).

(5) $$\text{Carry}-\text{over}\left(\%\right)=\left(m_{\mathrm{milk}}\times c_{AFM1}\right)/\left(m_{\mathrm{feed}}\times c_{AFB1}\right)\times 100$$

(6) $$\text{Carry}-\text{over}\left(\%\right)=0.5154\times e^{0.0521Y}\left(Y=\text{daily}\text{milk}\text{yield},\text{kg}\right)$$

(7) $$C_{\mathrm{milk}}\left(\text{ng}L^{-1}\right)=A\times \text{DMI}\times \left(\text{carry}-\text{over}/100\right)\times 1000/Y$$

To test whether Modules *A* and *C* are quantitatively reconcilable, predicted milk AFM_1_ was reconstructed from ration‐weighted feed AFB_1_ (Equation 7), where *A* is ration AFB_1_ (µg kg^−1^ DM), DMI is dry‐matter intake (kg day^−1^), and *Y* is daily yield (kg day^−1^). Three ration scenarios spanning Bangladeshi smallholder practice were evaluated (fodder‐based, typical mixed and maize‐heavy concentrate; Table 2), generating a predicted envelope that was compared with the distribution of observed dataset means. The comparison is a falsification test: an observed distribution lying largely outside the envelope would show that the two literatures cannot both be correct under the assumed transfer model, whereas agreement establishes only that they are compatible.

**TABLE 2:** Mechanistic reconstruction of milk AFM_1_ from reported feed AFB_1_ under three Bangladeshi ration scenarios (Equations 6 and 7). The comparison is a consistency check between two independent literatures and does not use paired feed–milk measurements (Sections 2.4 and 4.2).

Ration scenario	Ration AFB_1_ (µg kg^−1^ DM)	DMI (kg day^−1^)	Yield (kg day^−1^)	Carry‐over (%)	AFB_1_ intake (µg day^−1^)	Predicted milk AFM_1_ (ng L^−1^)
Low (fodder‐based ration)	4.0	7.0	10.0	0.87	28.0	24
Central (typical smallholder)	10.2	8.0	8.0	0.78	81.6	80
High (maize‐heavy concentrate)	30.0	8.0	6.0	0.70	240.0	282
Observed, for comparison	—	—	—	—	—	GM 92; weighted mean 143; range 6.6–699

*Note*: Predicted envelope 24–282 ng L^−1^ contains 18 of 20 observed dataset means (90%). Reproducing the observed weighted mean requires a ration‐weighted AFB_1_ of ∼18 µg kg^−1^; reproducing the extreme Dhaka mean requires ∼89 µg kg^−1^.

Equation 6 is the exponential relationship reported by Britzi et al. (2013), fitted to 12 Israeli‐Holstein cows in mid‐ and late‐lactation dosed with approximately 86 µg AFB_1_ daily for 7 days, with carry‐over evaluated at steady state on days 3–7; the reported coefficient of determination is *r* ^2^ = 0.622, and those authors found a linear specification to fit less well. Standard errors on the two coefficients and residual diagnostics are not given in the source and could not be recomputed here, because the animal‐level pairs of yield and carry‐over are unpublished; the relationship is therefore used as published rather than refitted. Its parameters, goodness of fit, calibration data and the carry‐over predicted across the milk‐yield range are tabulated in Table S7.

Two features of its provenance bound its use. First, the calibration data lie in the high‐yield region: the reported group mean carry‐over rates of 5.8% and 2.5% correspond, under the fitted function, to daily yields of approximately 46 and 30 kg, and the worked example in the source paper takes an early‐lactation cow at 45 kg day^−1^. Applying the function at the 5–15 kg day^−1^ yields typical of Bangladeshi smallholder cattle is therefore an extrapolation below the calibration range rather than an interpolation within it, and is reported here as such. Second, the extrapolated values remain consistent with the independent literature: the function returns 0.67%–1.13% across 5–15 kg day^−1^, against a reviewed central estimate of 1%–2% for dairy cows generally, rising to about 6% only at high production (Zentai et al. 2023). The extrapolation therefore predicts less transfer than the pooled literature would imply, which makes the consistency check of Section 3.5 harder rather than easier to pass.

Because the fit rests on few animals and on no Bangladeshi observation, coefficient uncertainty is not represented as a confidence band; it is propagated structurally in Section 3.5 by evaluating three ration scenarios and reporting the resulting envelope. No uncertainty from Equation 6 enters the exposure assessment, which is computed from the milk module alone (Equation 8); the role of the carry‐over model is confined to the consistency check, in which an erroneous coefficient would appear as a failure of the observed dataset means to fall within the predicted envelope.

### 2.5 Dietary Exposure and Risk Characterisation (Deterministic)

Chronic exposure was estimated as the EDI (Equation 8), the product of AFM_1_ concentration C (ng L^−1^) and Bangladesh‐specific milk intake IR (L day^−1^), normalised to body weight BW. Five age strata were modelled, with intakes reflecting the comparatively low Bangladeshi dairy consumption (adults 0.10 L day^−1^; infants 0.25 L day^−1^). Risk was characterised by the MOE (Equation 9) against an AFB_1_ BMDL_10_ (benchmark dose lower confidence limit [10%]) of 400 ng kg^−1^ bw day^−1^, an MOE below 10,000 denoting a public‐health concern for a genotoxic carcinogen (EFSA Scientific Committee 2005); by the HI (Equation 10) against a reference dose of 0.2 ng kg^−1^ bw day^−1^; and by the incremental HCC incidence (Equation 11), using an HBsAg‐weighted average potency (Bangladeshi carrier prevalence ≈ 5.4%) and an AFM_1_ relative potency factor of 0.1. Two deterministic scenarios were run: a central scenario using the sample‐weighted mean of the pooled datasets (143 ng L^−1^) and a high scenario (250 ng L^−1^) approximating the upper range of divisional raw‐milk means.

(8) EDI=(C×IR)/BW

(9) $$\text{MOE}={\text{BMDL}}_{10}/\text{EDI}$$

(10) $$\text{HI}=\text{EDI}/\text{RfD},\text{RfD}=0.2\;\text{ng}{\text{kg}}^{-1}\text{bw}{\text{day}}^{-1}$$

(11) $$\text{HCC}=\text{EDI}\times P_{\mathrm{avg}}\times 0.1,P_{\mathrm{avg}}=h_{+}\times 0.3+\left(1-h_{+}\right)\times 0.01$$

### 2.6 Probabilistic Exposure Modelling

Because deterministic point estimates conceal the variability of concentration, intake and body weight, a second‐order Monte Carlo simulation was implemented (100,000 iterations; seed fixed for reproducibility). Milk AFM_1_ was represented by a lognormal distribution fitted to the sample‐weighted distribution of dataset means (meanlog 4.524, sdlog 0.924; geometric mean 92 ng L^−1^, GSD 2.5); milk intake by a lognormal distribution with the age‐specific mean and a coefficient of variation of 35%; and body weight by a normal distribution with a coefficient of variation of 12%, truncated below at 40% of the mean. The three inputs were treated as independent, an assumption discussed in Section 4.5. Outputs are reported as the median and fifth percentile of the MOE, the proportion of iterations below the MOE benchmarks of 10,000 and 100 and the median HI (Table 3).

**TABLE 3:** Probabilistic dietary exposure and risk for AFM_1_ from milk (Monte Carlo, 100,000 iterations; lognormal concentration, meanlog 4.524, sdlog 0.924; intake CV 35%; body‐weight CV 12%).

Age group	EDI P50 (ng kg^−1^ bw day^−1^)	EDI P95 (ng kg^−1^ bw day^−1^)	MOE P50	MOE P05	MOE < 10,000 (%)	MOE < 100 (%)	HI P50
Infants (0.5–1 years)	2.55	13.46	157	30	100.0	32.8	12.8
Toddlers (1–3 years)	1.40	7.21	286	55	100.0	14.6	7.0
Children (4–9 years)	0.65	3.36	612	119	99.7	3.5	3.3
Adolescents (10–17 years)	0.23	1.18	1720	340	96.2	0.2	1.2
Adults (≥ 18 years)	0.15	0.74	2735	543	90.4	0.0	0.7

*Note*: Median MOEs exceed the deterministic values of Table 7 because the fitted distribution is centred on the geometric mean (92 ng L^−1^) rather than the sample‐weighted arithmetic mean (143 ng L^−1^); see Section 4.5.

### 2.7 Sensitivity, Robustness and Structured Uncertainty Analysis

Small‐study effects were assessed by funnel‐plot inspection, Egger's regression intercept test (Egger et al. 1997), Begg's rank correlation and the Duval–Tweedie trim‐and‐fill procedure (Duval and Tweedie 2000). Robustness of the pooled prevalence was examined by leave‐one‐out re‐estimation of all 20 datasets (Table S3). Robustness of the exposure estimate was examined by (i) removing the single most influential high‐concentration dataset, (ii) restricting the analysis to fully referenced peer‐reviewed datasets, (iii) applying 20% and 50% downward corrections for possible ELISA overestimation relative to chromatographic confirmation and (iv) varying the BMDL_10_ between 250 and 600 ng kg^−1^ bw day^−1^. A one‐at‐a‐time tornado analysis quantified the influence of each input on the infant MOE (Figure 7A).

DerSimonian–Laird was pre‐specified as the primary estimator for comparability with the existing prevalence meta‐analysis literature. Because it is known to underestimate *τ* ^2^, and hence to yield anticonservative CIs, when heterogeneity is moderate, the pooled prevalence was re‐estimated under restricted maximum likelihood (REML) and under the Paule–Mandel estimator, and the Hartung–Knapp adjustment (which replaces the normal approximation with a t distribution and an inflated variance term) was applied to each. These results are reported in Section 3.8 as an estimator sensitivity analysis rather than as competing primary estimates.

Finally, uncertainty was tabulated following the EFSA guidance on uncertainty analysis in scientific assessments (EFSA Scientific Committee 2018). Each identified source was classified by nature (parameter, model or scenario uncertainty), by the expected direction of its effect on the risk estimate, by its approximate magnitude and by whether, acting alone, it could plausibly reverse the study's principal conclusion (Table 4). Computations used Python 3.12 (NumPy, SciPy), with *α* = 0.05.

**TABLE 4:** Structured uncertainty analysis of the risk estimates, classified by nature, direction and magnitude following EFSA guidance (EFSA Scientific Committee 2018). The final column indicates whether the source, acting alone at its plausible extreme, could raise the infant MOE above the 10,000 benchmark.

SL. No.	Source of uncertainty	Nature and direction	Approximate magnitude and how addressed	Could it reverse the conclusion?
1	Choice of central tendency for milk AFM_1_ (arithmetic 143 vs. geometric 92 ng L^−1^)	Parameter; either direction	Infant MOE 95 vs. 157 (1.7×); deterministic and probabilistic estimates reported side by side (Tables 3 and 7)	No
2	Influence of one high‐concentration Dhaka dataset	Parameter; overestimation	Weighted mean 143 → 93 ng L^−1^; infant MOE 95 → 146; leave‐one‐out and restricted analyses (Section 3.8)	No
3	ELISA overestimation relative to chromatographic confirmation	Measurement; overestimation	0%–50%; infant MOE 95 → 142; correction scenarios (Sections 3.7–3.8)	No
4	Six datasets at high risk of bias/uncertain provenance	Parameter; either direction	Excluding them raises the weighted mean to 172 ng L^−1^ (infant MOE 79); risk‐of‐bias subgroup analysis	No
5	Convenience rather than probability sampling in primary surveys	Scenario; unknown direction	Not quantifiable; stated as a limitation and representativeness qualified	Unknown, not bounded
6	Concentration data available only as dataset means	Model; understates spread	Understates upper exposure percentiles; probabilistic model acknowledged as conservative in shape only	No
7	Carry‐over coefficient extrapolated from high‐yielding non‐Bangladeshi cows	Model; either direction	Affects the feed–milk consistency check only; no effect on exposure estimates (Sections 2.4, 4.2)	Not applicable
8	Milk‐intake rates representative rather than individually surveyed	Parameter; either direction	Infant MOE 68–158 across 0.15–0.35 L day^−1^; tornado analysis (Figure 7A)	No
9	BMDL_10_ selection (250–600 ng kg^−1^ bw day^−1^)	Parameter; either direction	Infant MOE 59–142; tornado analysis (Figure 7A)	No
10	AFM_1_ relative potency factor of 0.1	Model; either direction	Affects HCC estimates proportionally; HCC treated as order‐of‐magnitude only	Not applicable (MOE unaffected)
11	HBsAg‐weighted potency assumes independence of carrier status and milk intake	Model; either direction	Affects HCC estimates only; stated as a limitation	Not applicable (MOE unaffected)
12	Independence of concentration, intake and body weight in the Monte Carlo model	Model; overestimates extremes	Slight inflation of tail percentiles; medians unaffected	No
13	Clustering of 20 datasets within a smaller number of reports	Model; understates variance	Confidence interval slightly too narrow; prediction intervals reported throughout	No
14	Undetected reporting or publication bias (tests underpowered at *k *= 20)	Model; either direction	Trim‐and‐fill imputes one dataset; immaterial (Section 3.8)	No

*Note*: Overturning the principal conclusion would require simultaneous, same‐direction error of roughly two orders of magnitude across sources 1–4, 8 and 9; no single source, and no plausible combination of those identified, achieves this.

## 3 Results

### 3.1 Included Studies and the Structure of the Evidence Base

The searches returned 318 records; after deduplication and screening, 12 reports were eligible and provided 20 region‐, site‐ or milk‐type‐specific datasets comprising 605 milk samples (Figure 2). The datasets span 10 divisions and areas together with national samples, and cover raw, pasteurised and UHT milk (Table 1). All used competitive ELISA and none reported chromatographic confirmation. Under the prevalence‐adapted Newcastle–Ottawa appraisal, nine datasets were classified at low, five at moderate and six at high risk of bias. The 20 datasets derive from 10 sources, four of which contribute multiple strata; the datasets are therefore clustered rather than independent, and this is carried through the weighting, the sensitivity analyses of Section 3.8 and the interpretation in Section 4.3.

### 3.2 Pooled Prevalence of AFM_1_ in Milk, and the Structure of Its Heterogeneity

The random‐effects pooled prevalence of AFM_1_ in Bangladeshi milk was 58.7% (95% CI 51.6%–65.5%), with moderate‐to‐high heterogeneity (*Q* = 49.9, df = 19, *p* < 0.001; *τ* ^2^ = 0.253; *I* ^2^ = 61.9%) (Figure 3, Table 5). The 95% prediction interval, however, was 32.1%–81.1%. This is the more policy‐relevant statistic: the CI states how precisely the average of the existing literature has been located, whereas the prediction interval states the range within which the prevalence of a new Bangladeshi survey would be expected to fall. Its width indicates that a national point prevalence should not be treated as a stable planning figure.

**FIGURE 3:**
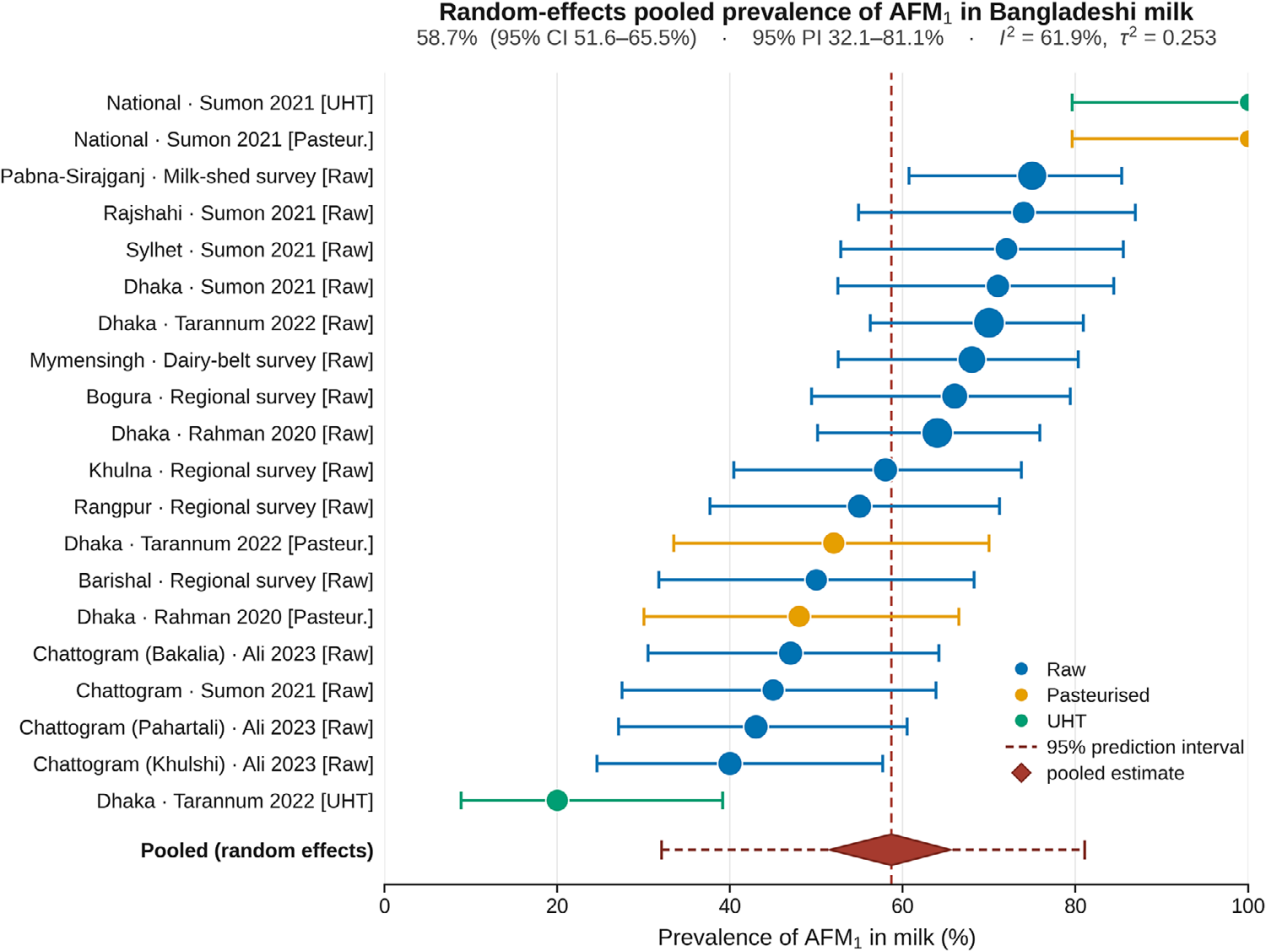
Forest plot of the random‐effects pooled prevalence of AFM_1_ in Bangladeshi milk by division, study and milk type. Point areas are proportional to sample size; colour denotes milk type; bars are 95% Wilson score intervals; the red diamond is the overall estimate (58.7%, 95% CI 51.6%–65.5%; 95% prediction interval 32.1%–81.1%; *I* ^2^ = 61.9%, *τ* ^2^ = 0.253).

**TABLE 5:** Random‐effects pooled prevalence of AFM_1_ in Bangladeshi milk, overall and by pre‐specified subgroup, with 95% prediction intervals (PI), heterogeneity statistics and tests of between‐subgroup difference (*Q*‐between).

Stratum	*k*	Samples	Pooled prev. % (95% CI)	95% PI (%)	*τ* ^2^	*I* ^2^ (%)	*Q*‐between (*p*)
Overall	20	605	58.7 (51.6–65.5)	32.1–81.1	0.253	61.9	—
By milk type							0.04 (0.980)
Raw	15	500	60.4 (54.1–66.4)	40.5–77.4	0.123	47.8	
Pasteurised	3	65	59.0 (33.7–80.3)	0–100	0.471	61.9	
UHT	2	40	69.5 (2.0–99.6)	Not estimable	10.391	89.4	
By region							13.49 (0.001)
Dhaka	6	203	55.3 (41.0–68.9)	15.9–88.6	0.378	73.1	
Chattogram	4	115	43.7 (34.9–52.9)	26.4–62.6	0.000	0.0	
Other divisions/national	10	287	66.8 (58.7–74.1)	44.9–83.4	0.120	39.9	
By risk of bias							0.92 (0.339)
Low/moderate	14	400	57.0 (47.0–66.5)	25.4–83.5	0.371	68.5	
High	6	205	63.1 (55.3–70.2)	46.8–76.9	0.031	19.0	

*Note*: Division‐level estimates for single‐dataset divisions are given in Table S2. The UHT subgroup (*k *= 2, prevalences 20% and 100%) is not interpretable and is shown only for completeness.

Region, not milk type, structured the heterogeneity (Table 5). Prevalence differed significantly between regions (*Q*‐between = 13.5, df = 2, *p* = 0.001), from 43.7% in Chattogram (*k* = 4; *I* ^2^ = 0.0%, *p* = 0.956) to 55.3% in Dhaka (*k* = 6), where between‐dataset inconsistency was greatest (*I* ^2^ = 73.1%), and 66.8% in other divisions and national samples (*k* = 10). Raw, pasteurised and UHT milk did not differ (*Q*‐between = 0.04, df = 2, *p* = 0.980; raw 60.4% vs. processed 59.4%, *p* = 0.945), consistent with the thermal stability of AFM_1_. Datasets classified as at high risk of bias returned a slightly higher prevalence than fully referenced peer‐reviewed datasets (63.1% vs. 57.0%), but the difference was not significant (*p* = 0.339) and excluding them left the pooled estimate essentially unchanged. Exploratory meta‐regression found no significant moderating effect of mean concentration (*β *= 0.31, *p* = 0.405), dataset size (*β *= 0.020, *p* = 0.232) or raw‐milk status (*β *= 0.42, *p *= 0.292); residual *I* ^2^ remained 21%–23% in each single‐covariate model, so a substantial part of the between‐dataset variability is not explained by any measured moderator and is most plausibly attributable to unmeasured differences in sampling frame, season of collection, and ELISA kit and cut‐off.

Of the three pre‐specified subgroup comparisons, only region remained significant after Holm adjustment for multiplicity (nominal *p* = 0.001, adjusted *p* = 0.004). Neither milk type (nominal *p* = 0.980, adjusted *p* = 0.980) nor the three‐level risk‐of‐bias comparison (*Q*‐between = 5.44, df = 2, nominal *p* = 0.066, adjusted *p* = 0.131) survived correction. The regional difference is therefore the only subgroup finding that withstands adjustment, and even that is reported as hypothesis‐generating rather than as an established regional gradient.

### 3.3 Concentration Distribution and the Exceedance Profile

Concentrations were strongly right‐skewed and are therefore summarised by more than one statistic. The sample‐weighted arithmetic mean across datasets was 143 ng L^−1^, the unweighted mean 118 ng L^−1^, the median of dataset means 88 ng L^−1^ and the sample‐weighted geometric mean 92 ng L^−1^ (GSD 2.5). Typical raw‐milk means lay between 35 and 120 ng L^−1^, but the Dhaka raw‐milk survey of Tarannum et al. (2022) reported a mean near 699 ng L^−1^; removing this single dataset lowered the weighted mean to 93 ng L^−1^, a 35% reduction produced by 8% of the samples. Pasteurised and UHT milk carried 35–106 ng L^−1^.

The dispersion of the dataset means is as informative as their centre. The interquartile range was 57–111 ng L^−1^ against a full range of 6.6–699.1 ng L^−1^, so the middle half of the literature spans a factor of two while the whole spans a factor of more than one hundred; the median dataset prevalence was 61.0% (IQR 47.8%–71.2%). The arithmetic mean is accordingly governed by the upper tail rather than by the central body of the distribution, which is why every downstream exposure estimate is reported for both an arithmetic and a geometric central tendency (Sections 3.6 and 3.7).

Rather than replot these means, Figure 4A presents the exceedance profile of the pooled sample, the proportion of sampled milk lying above any given threshold. On this basis, 83% of sampled milk exceeded the EU ML of 50 ng L^−1^, and 8% exceeded the BSTI/BFSA and Codex limit of 500 ng L^−1^ (European Commission 2006; FAO/WHO Joint Expert Committee on Food Additives (JECFA) 2018; Bangladesh Food Safety Authority 2017). Figure 4B shows that prevalence and concentration are largely decoupled across datasets: the two datasets reporting 100% positivity carried among the lowest mean concentrations, whereas the highest concentration was recorded at a prevalence close to the pooled value. Prevalence and mean concentration are therefore not interchangeable indicators of risk, and the exposure assessment depends on the concentration distribution rather than on the pooled prevalence.

**FIGURE 4:**
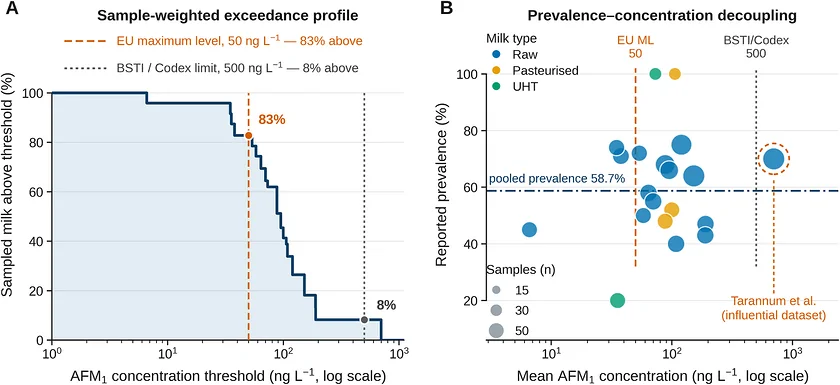
Distributional analysis of milk AFM_1_. (A) Sample‐weighted exceedance profile: the proportion of sampled milk above each concentration threshold, with the EU maximum level (50 ng L^−1^; 83% of samples above) and the BSTI/BFSA/Codex limit (500 ng L^−1^; 8% above) marked. (B) Prevalence plotted against mean concentration by dataset, showing that the two indicators are largely decoupled (exploratory meta‐regression *β *= 0.31, *p* = 0.405); symbol area is proportional to sample size, colour denotes milk type and the influential Dhaka dataset of Tarannum et al. is ringed.

### 3.4 Aflatoxins and Mycotoxins in Animal Feed

Bangladeshi surveys place AFB_1_ at 0–160 µg kg^−1^ in poultry feed and 1–103 µg kg^−1^ in maize, with both matrices frequently exceeding the 20 µg kg^−1^ feed ML, while compound beef‐cattle feed (7.8–14.7 µg kg^−1^) and ingredients such as oil cakes, bran, rice straw and soybean meal averaged approximately 10–13 µg kg^−1^ (Table 6). Although the cattle‐feed means largely satisfy the 20 µg kg^−1^ limit, they uniformly exceed the stricter EU dairy‐feed limit of 5 µg kg^−1^, implying a persistent baseline of carry‐over pressure even where feed is nominally compliant. Maize additionally co‐occurs with fumonisins (up to 9.18 mg kg^−1^) and with zearalenone and sterigmatocystin in dairy feed and forage, indicating a multi‐mycotoxin burden that the single‐toxin risk metrics used here do not capture. These data are reported descriptively and were not pooled: the underlying surveys differ in matrix definition, sampling frame and analytical method, and most do not report the sample‐level distributions that meta‐analysis would require. Both feed AFB_1_ and milk AFM_1_ show a June–August monsoon peak (Figure 5), when storage humidity maximises fungal growth.

**TABLE 6:** Aflatoxin B_1_ and co‐occurring mycotoxins reported in Bangladeshi animal feeds and ingredients, with compliance against the 20 µg kg^−1^ general feed maximum level and the 5 µg kg^−1^ EU limit for dairy‐cattle feed.

Feed/ingredient	Source	AFB_1_ range (µg kg^−1^)	Mean (µg kg^−1^)	Exceeds 20 µg kg^−1^ ML	Exceeds 5 µg kg^−1^ (EU dairy)	Co‐occurring toxins reported
Poultry feed	Lubna et al. (2018), Naveed et al. (2022)	0–160	∼38	Yes (mean and upper range)	Yes	Not reported
Maize	Roy et al. (2022)	1.07–103	∼28	Yes (mean and upper range)	Yes	Fumonisins (to 9.18 mg kg^−1^), zearalenone
Compound cattle feed	Kamal et al. (2022)	7.78–14.66	∼11	No	Yes	Not reported
Oil cakes/bran	Kamal et al. (2022)	9.0–16	∼12	No	Yes	Not reported
Rice straw (fodder)	Phillips et al. (1996)	∼12.05	∼12	No	Yes	Sterigmatocystin, zearalenone
Soybean meal	Kamal et al. (2022)	4–15	∼9.8	No	Yes (mean)	Not reported

*Note*: Reported descriptively; not pooled (Section 3.4).

**FIGURE 5:**
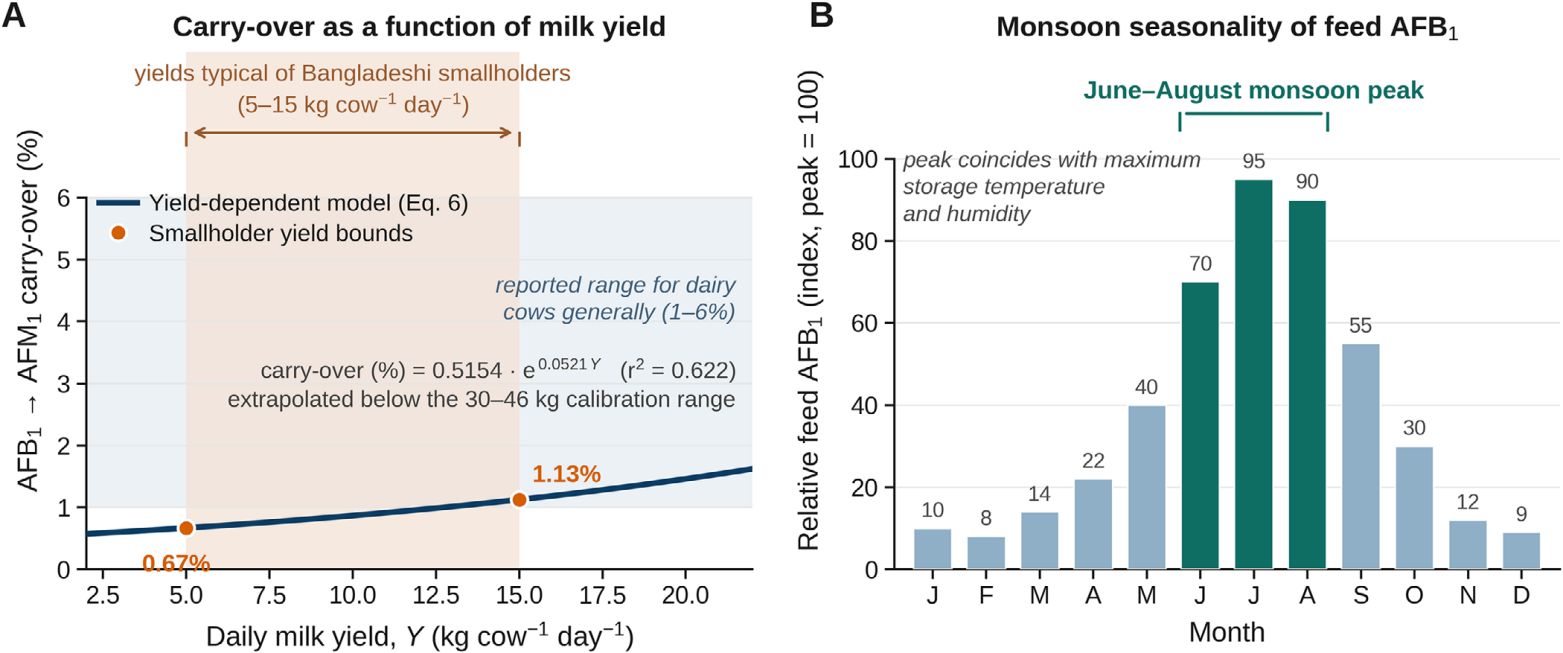
(A) Feed‐to‐milk carry‐over as a function of daily milk yield under Equation 6, returning 0.67%–1.13% across the 5–15 kg cow^−1^ day^−1^ yields typical of Bangladeshi smallholders (marked); the shaded horizontal band is the range reported for dairy cows generally. Application below the 30–46 kg calibration range is an extrapolation, and no confidence band is shown because the animal‐level calibration data are unpublished (Section 2.4). (B) Monsoon seasonality of feed AFB_1_, peaking June–August. The two series derive from independent studies and years; the shared seasonal maximum is consistent with a common humidity‐driven mechanism but is not evidence of coupling within a single production system.

Because the feed and milk series are drawn from different studies, sites and years, this shared maximum is reported as a coincidence of pattern between two independently sampled literatures. It is consistent with a common humidity‐driven mechanism, but it does not establish that monsoon feed contamination produces the milk residues observed, and no causal weight is placed on it in the risk characterisation.

### 3.5 Mechanistic Consistency Between the Feed and Milk Modules

Applying the yield‐dependent carry‐over function (Equation 6) to the low daily yields typical of Bangladeshi smallholder cattle gives transfer of 0.67%–1.13% at 5–15 kg day^−1^. Reconstructed milk AFM_1_ (Equation 7) across the three ration scenarios spans 24–282 ng L^−1^, with a central prediction of 80 ng L^−1^ (Table 2, Figure 6A). This envelope contains 18 of the 20 observed dataset means (90%), and the central prediction lies close to the observed geometric mean of 92 ng L^−1^ (Figure 6B). Reproducing the observed sample‐weighted mean of 143 ng L^−1^ requires a ration‐weighted AFB_1_ of approximately 18 µg kg^−1^, a value that lies between the reported cattle‐feed mean (∼10 µg kg^−1^) and the maize mean (∼28 µg kg^−1^) and is therefore attainable in maize‐inclusive rations. Reproducing the extreme Dhaka mean of 699 ng L^−1^ would require approximately 89 µg kg^−1^, close to the upper end of the reported feed range and reached in the surveyed literature only by poultry feed and by the most contaminated maize.

**FIGURE 6:**
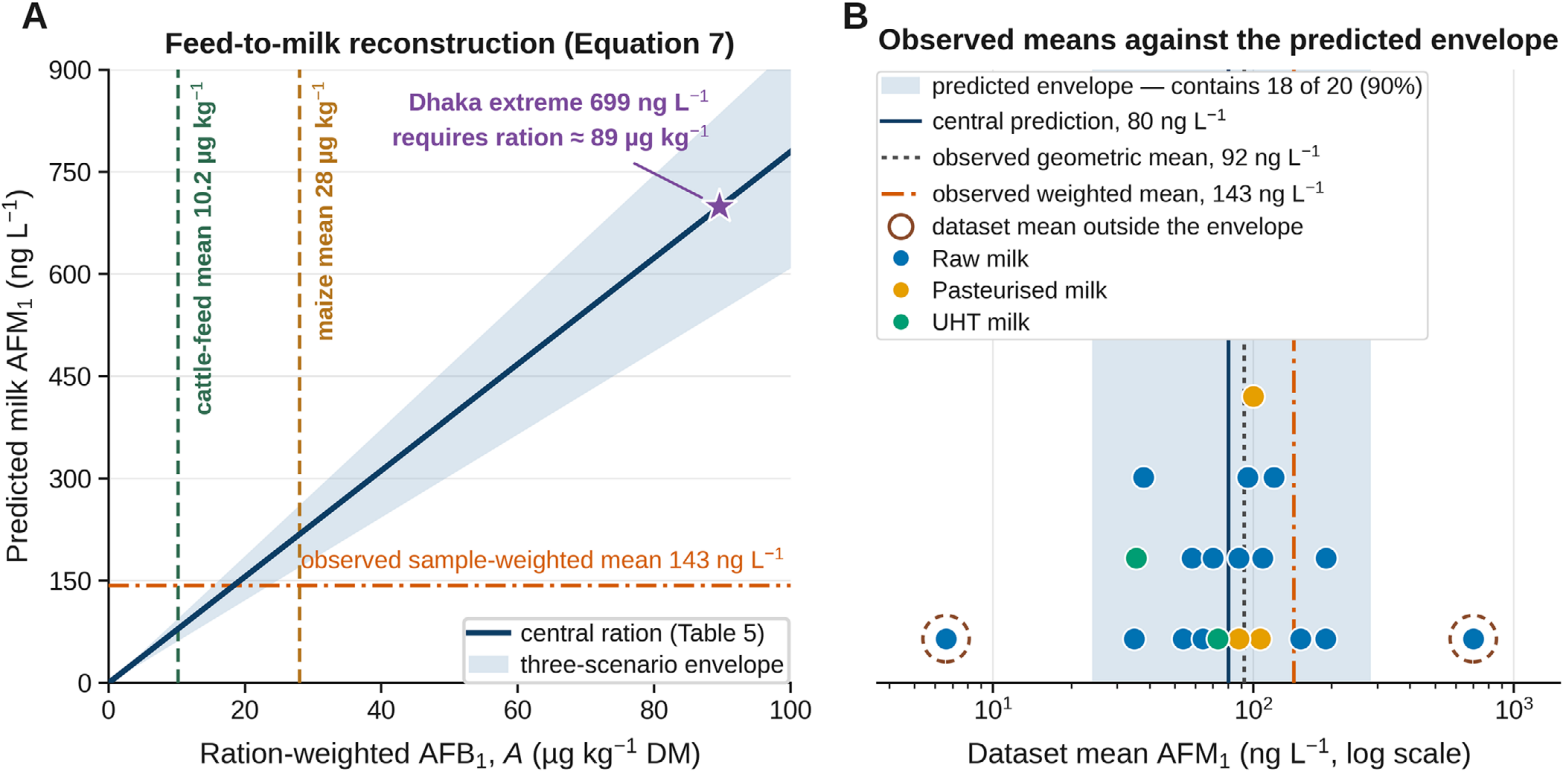
Mechanistic consistency check between the feed and milk modules. (A) Predicted milk AFM_1_ as a function of ration‐weighted AFB_1_ under the yield‐dependent carry‐over model (Equations (6), (7)), with the envelope spanning the three ration scenarios of Table 2 and the reported feed means and observed milk means marked. (B) The 20 observed dataset means shown individually against the predicted envelope, which contains 18 of 20 (90%); the two means falling outside it (6.6 and 699.1 ng L^−1^) are ringed. The analysis uses no paired feed–milk measurements and tests compatibility, not transfer (Section 4.2).

Two datasets fall outside the envelope (the very low Chattogram mean of 6.6 ng L^−1^ and the extreme Dhaka mean of 699 ng L^−1^), so the tails of the observed distribution are not explained by a single central ration scenario. The two literatures are thus quantitatively compatible under published transfer parameters, which would not be the case if either were grossly biased; the analysis uses no paired measurements and is interpreted in Section 4.2.

### 3.6 Deterministic Dietary Exposure and Risk

Using the sample‐weighted mean of 143 ng L^−1^, EDIs decreased steeply with age, from 4.22 ng kg^−1^ bw day^−1^ in infants to 0.24 in adults (Table 7). Every age group returned an MOE far below the 10,000 benchmark (95 for infants, 175 for toddlers, 372 for children, 1055 for adolescents and 1649 for adults), and the HI exceeded unity for all groups (21.1 in infants to 1.2 in adults). The incremental HCC incidence peaked at 0.0108 cases year^−1^ per 100,000 infants. Under the high scenario (250 ng L^−1^), the infant MOE fell to 54.

**TABLE 7:** Deterministic age‐stratified dietary exposure (EDI) and risk (MOE, HI, HCC) for AFM_1_ from milk in Bangladesh under central (143 ng L^−1^) and high (250 ng L^−1^) scenarios. Probabilistic counterparts are given in Table 3. MOE < 10,000 and HI > 1 denote concern.

Age group	Scenario	EDI (ng kg^−1^ bw day^−1^)	MOE	HI	HCC
Infants (0.5–1 years)	Central	4.22	95	21.1	0.0108
	High	7.35	54	36.8	0.0189
Toddlers (1–3 years)	Central	2.29	175	11.4	0.0059
	High	4.00	100	20.0	0.0103
Children (4–9 years)	Central	1.08	372	5.4	0.0028
	High	1.88	213	9.4	0.0048
Adolescents (10–17 years)	Central	0.38	1055	1.9	0.0010
	High	0.67	600	3.3	0.0017
Adults (≥ 18 years)	Central	0.24	1649	1.2	0.0006
	High	0.42	960	2.1	0.0011

*Note*: HCC in incremental cases year^−1^ per 100,000 persons. All MOE values fall below 10,000. Intakes and body weights used: infants 0.25 L day^−1^/8.5 kg; toddlers 0.20/12.5; children 0.15/20.0; adolescents 0.12/45.0; adults 0.10/60.0.

### 3.7 Probabilistic Exposure and One‐at‐a‐Time Sensitivity

The Monte Carlo simulation reproduced the direction of the deterministic result while widening its interval and shifting its centre (Table 3, Figure 7B). Median MOEs were 157 (infants), 286 (toddlers), 612 (children), 1720 (adolescents) and 2735 (adults); fifth‐percentile MOEs were 30, 55, 119, 340 and 543, respectively. The median values exceed the corresponding deterministic estimates because the lognormal concentration distribution is centred on the geometric mean (92 ng L^−1^) rather than the arithmetic mean (143 ng L^−1^), a divergence that follows from the skew documented in Section 3.3 and that a deterministic assessment cannot express. The proportion of iterations returning MOE < 10,000 was 100% for infants and toddlers, 99.7% for children, 96.2% for adolescents and 90.4% for adults; the proportion returning MOE < 100 was 32.8% for infants and 14.6% for toddlers. Median HIs remained above unity for infants (12.8), toddlers (7.0), children (3.3) and adolescents (1.2), but not for adults (0.7), a divergence from the deterministic result attributable entirely to the choice of central tendency, discussed in Section 4.5.

**FIGURE 7:**
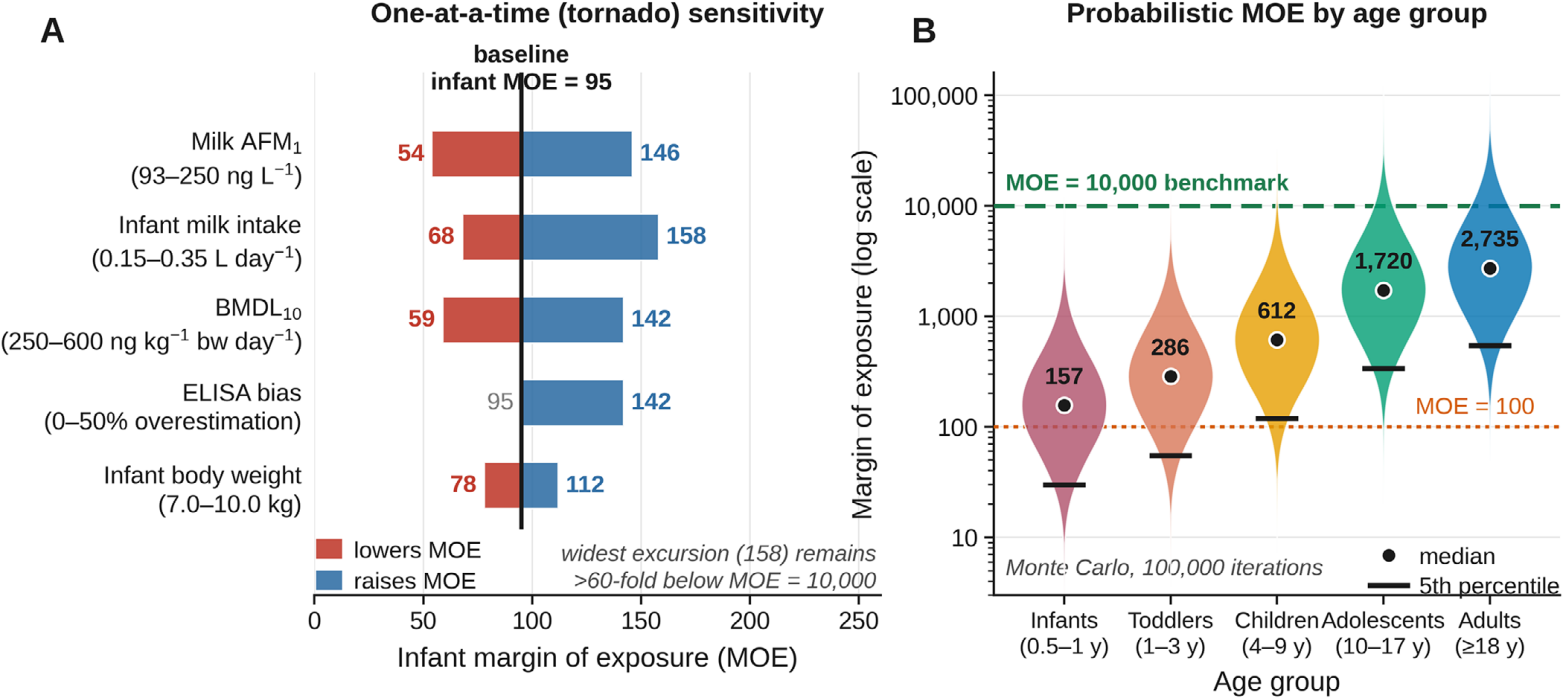
Uncertainty and sensitivity of the risk estimates. (A) One‐at‐a‐time (tornado) analysis of the infant MOE against a baseline of 95, showing that no single input perturbation raises it above 158, more than 60‐fold below the benchmark. (B) Probabilistic MOE distributions by age group (Monte Carlo, 100,000 iterations); dots are medians, bars are fifth percentiles, and the dashed line marks the MOE = 10,000 benchmark, which no age group approaches.

The one‐at‐a‐time analysis (Figure 7A) shows that no single input governs the conclusion. Against a baseline infant MOE of 95, varying the milk concentration across its plausible range (93–250 ng L^−1^) moved the MOE to 54–146; varying infant milk intake (0.15–0.35 L day^−1^) to 68–158; varying the BMDL_10_ (250–600 ng kg^−1^ bw day^−1^) to 59–142; correcting for 0%–50% ELISA overestimation to 95–142; and varying infant body weight (7.0–10.0 kg) to 78–112. The widest excursion remains more than 60‐fold below the MOE = 10,000 benchmark.

### 3.8 Robustness, Small‐Study Effects and the Stability of the Principal Conclusion

Egger's regression showed no significant small‐study effect (intercept 1.23, SE 1.38, *t* = 0.89, *p* = 0.385), Begg's rank correlation was non‐significant (*τ* = 0.064, *p* = 0.697), the funnel plot was symmetric (Figure 8) and trim‐and‐fill imputed one missing dataset, whose inclusion would not materially alter the pooled estimate. These tests have low power at *k* = 20, and, for prevalence data, funnel asymmetry is a weak indicator of reporting bias; the absence of detected asymmetry is therefore reassuring but not probative.

**FIGURE 8:**
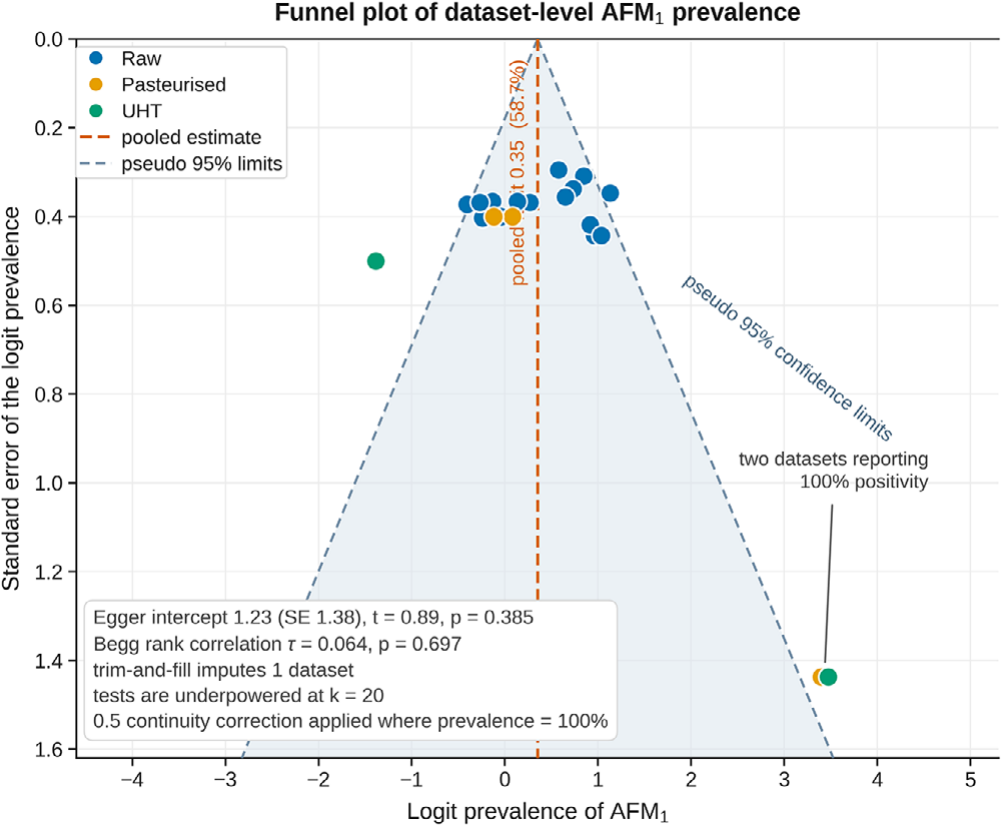
Funnel plot of dataset‐level AFM_1_ prevalence against standard error (logit scale; a 0.5 continuity correction is applied to the two datasets reporting 100% positivity, which coincide and are separated here by a small horizontal offset). Symmetry, a non‐significant Egger intercept test (*p *= 0.385), a non‐significant Begg rank correlation (*p* = 0.697) and a trim‐and‐fill imputation of one dataset argue against substantial small‐study effects, although these tests are underpowered at *k* = 20.

Leave‐one‐out re‐estimation moved the pooled prevalence only between 57.6% and 60.4% (Table S3). The exposure estimate proved less stable, and its sensitivity is reported in full because it drives the risk conclusion: excluding the influential Dhaka dataset gave a weighted mean of 93 ng L^−1^ and an infant MOE of 146; restricting the analysis to fully referenced peer‐reviewed datasets gave 172 ng L^−1^ and an infant MOE of 79; applying both restrictions gave 97 ng L^−1^ and 140; and correcting for 50% ELISA overestimation gave 96 ng L^−1^ and 142. Across every combination examined, and across the full probabilistic distribution, the infant MOE remained between 30 and 158, that is, between 60‐ and 330‐fold below the benchmark of 10,000, and the infant HI remained above 12. The principal conclusion is therefore robust to every individual analytical choice tested, even though the numerical value of the exposure estimate is not.

Alternative heterogeneity estimators left the pooled prevalence essentially unchanged. The estimate was 58.7% (95% CI 51.6%–65.5%) under DerSimonian–Laird (*τ* ^2^ = 0.253), 58.7% (51.8%–65.3%) under REML (*τ* ^2^ = 0.227) and 59.0% (50.5%–67.0%) under Paule–Mandel (*τ* ^2^ = 0.423). The Hartung–Knapp adjustment widened the interval without moving the estimate, to 50.2–66.7% with REML and 49.9%–67.6% with Paule–Mandel. Across all five specifications, the point estimate varies by less than 0.4 percentage points, while the CI widens by up to 3.5 percentage points. Paule–Mandel returns the largest *τ* ^2^ and correspondingly the widest prediction interval (25.9%–85.6%), which strengthens rather than weakens the conclusion of Section 3.2 that a single national prevalence is not a stable planning figure.

The two unit‐of‐analysis sensitivity analyses addressed the clustering of strata within reports. Summing positives and denominators within each of the 10 sources gave a pooled prevalence of 60.3% (95% CI 52.6%–67.5%; *τ* ^2^ = 0.167; *I* ^2^ = 68.9%), and retaining only the largest stratum from each source gave 63.3% (57.5%–68.7%; *I* ^2^ = 19.3%). Both lie within five percentage points of the primary estimate, so the substantive finding does not depend on the treatment of clustered strata. The aggregated analysis nevertheless returned a wider CI than the primary analysis on the same 605 samples, confirming that the *k* = 20 specification credits the evidence base with more independent information than it holds. The primary estimate is therefore best read as resting on 10 sources rather than 20 independent observations, and its CI as a lower bound on the true uncertainty.

## 4 Discussion

### 4.1 Principal Findings

Three findings carry the assessment. Pooled prevalence is high but imprecisely located: 58.7% of milk sampled to date carried detectable AFM_1_, but the prediction interval of 32.1%–81.1% shows that this figure describes a heterogeneous literature rather than a stable descriptor of Bangladeshi milk. Risk is driven by the concentration distribution rather than by prevalence: it is strongly right‐skewed, sensitive to a single influential dataset, and places 83% of sampled milk above the EU ML. And modelled margins of exposure for infants and young children fall between 16‐ and 330‐fold below the MOE = 10,000 benchmark under every scenario, restriction and correction examined, which is the finding on which the policy implications rest.

### 4.2 What This Evidence Architecture Can and Cannot Support

The design of this assessment is modular and literature‐based, and its inferential reach is bounded accordingly. Because no Bangladeshi study measures AFB_1_ in feed and AFM_1_ in milk in the same animals, farms or sampling frame, the two modules are joined by a carry‐over coefficient transferred from high‐yielding cows under controlled feeding outside Bangladesh (Britzi et al. 2013), not by any measured within‐system association. The consistency analysis of Section 3.5 should therefore be read for exactly what it is: a demonstration that two independently assembled literatures are quantitatively compatible under an explicit transfer model, with 90% of observed dataset means falling inside the predicted envelope. Such compatibility is meaningful and falsifiable, since gross bias in either literature would have produced a mismatch, but it is a coherence test rather than evidence of causal transfer, and it does not license attributing any observed milk residue to any observed feed sample.

The same boundary applies to the seasonal argument. The shared June–August peak in feed AFB_1_ and milk AFM_1_ is consistent with a common humidity‐driven mechanism, but the two series are drawn from different studies, sites and years, and a shared maximum in independently sampled series is equally compatible with parallel but unlinked contamination. Human exposure here is driven exclusively by the milk module, and the risk estimates would be unchanged if the feed module were removed; the contribution of the feed module is to show that the observed milk residues are mechanistically plausible and to identify where intervention would be most efficient.

### 4.3 Methodological Limitations of the Underlying Evidence

Four features of the primary literature constrain the reliability of the pooled estimates. First, measurement: every included dataset used competitive ELISA without chromatographic confirmation. ELISA is prone to overestimation through matrix effects and cross‐reactivity with structurally related metabolites, and comparative studies typically report positive bias relative to HPLC‐FLD or LC‐MS/MS. The 50% correction scenario of Section 3.8 was included for this reason; it lowers the weighted mean to 96 ng L^−1^ but leaves the infant MOE at 142. Second, censoring and reporting: limits of detection and recovery were reported inconsistently, dataset means were generally reported without dispersion, and the treatment of left‐censored values is not recoverable from most reports. The concentration distribution used in the probabilistic model was therefore fitted to dataset means rather than to sample‐level data, which understates within‐dataset variability and, correspondingly, the true spread of individual exposures. Third, sampling: most surveys used convenience sampling of markets, collection points or farms rather than probability sampling of the national milk supply, so the pooled estimate represents the sampled locations rather than Bangladeshi milk as consumed. Fourth, provenance and independence: six of the 20 datasets are not attached to a fully referenced peer‐reviewed report and were classified as at high risk of bias, and four reports contribute multiple strata, so the effective number of independent observations is smaller than *k* = 20 implies. Excluding the high‐risk datasets did not change the qualitative conclusion, but it raised the weighted mean concentration to 172 ng L^−1^, showing that the direction of this bias is not obvious a priori.

### 4.4 Sources and Interpretation of Heterogeneity

Heterogeneity was moderate‐to‐high overall (*I* ^2^ = 61.9%, *τ* ^2^ = 0.253) and is best interpreted through its structure rather than its magnitude. Region was the only moderator with a significant effect (*Q*‐between = 13.5, *p* = 0.001), spanning 43.7% in Chattogram to 66.8% in other divisions; within Chattogram, the four datasets were entirely consistent (*I* ^2^ = 0.0%), whereas the six Dhaka datasets were the most inconsistent (*I* ^2^ = 73.1%), reflecting a metropolitan milk‐shed that aggregates milk of diverse provenance. Milk type explained nothing (*p* = 0.980), which is mechanistically expected given the thermal stability of AFM_1_ and indicates that the apparent difference between pasteurised and UHT milk in earlier descriptive accounts reflects sampling rather than processing. Exploratory meta‐regression left residual *I* ^2^ of 21%–23%, so a material component of between‐dataset variability remains unexplained by any recorded moderator; the most plausible unmeasured contributors are season of collection (unreported in most surveys), ELISA kit and declared cut‐off, and the position of the sampled milk in the supply chain. Two subgroup estimates should not be interpreted at all: the UHT subgroup rests on two datasets reporting 20% and 100% positivity, giving *τ* ^2^ = 10.4 and a prediction interval spanning the entire 0%–100% range.

### 4.5 Uncertainty and the Robustness of the Derived Risk Estimates

Table 4 sets out the uncertainty attaching to the risk estimates source by source, classified by nature, direction and magnitude following EFSA guidance (EFSA Scientific Committee 2018). Three points are central.

First, the largest single source is not statistical but parametric: the choice of central tendency for the milk concentration. The arithmetic mean (143 ng L^−1^), the geometric mean (92 ng L^−1^) and the mean excluding the influential Dhaka dataset (93 ng L^−1^) differ by more than 50%, and this choice alone moves the infant MOE between 95 and 157. The probabilistic result is centred on the geometric mean and is therefore less conservative than the deterministic result, the reverse of the usual expectation, and the reason the median adult HI falls below unity (0.7) in the probabilistic model while exceeding it (1.2) in the deterministic one. Neither is correct in preference to the other; they answer different questions, the deterministic estimate approximating a high‐consumption, average‐concentration scenario and the probabilistic median a typical consumer.

Second, several sources are model uncertainties whose magnitude cannot be bounded from the available data. The carry‐over function is extrapolated from high‐yielding cows to smallholder animals of very different nutritional and physiological status; the relative potency factor of 0.1 for AFM_1_ is an approximation derived from limited animal data; the HBsAg‐weighted potency assumes that carrier status and milk consumption are independent; and the Monte Carlo model treats concentration, intake and body weight as independent, whereas intake and body weight are positively correlated within age strata, a simplification that widens the simulated distribution and slightly inflates the extreme percentiles.

Third, and decisively for interpretation, these uncertainties do not act on the conclusion as they act on the numbers. The infant MOE ranged from 30 (fifth percentile, probabilistic) to 158 (most favourable single‐input perturbation) across every analysis performed, against a benchmark of 10,000. Overturning the conclusion would require the true exposure to be overestimated by roughly two orders of magnitude, and hence the concentration data, the intake data and the potency benchmark all to be wrong in the same direction and by large factors simultaneously; Table 4 records no single source, and no plausible combination of those identified, capable of that. The direction and public‐health significance of the finding are therefore robust, whereas the numerical value of the exposure estimate is uncertain by roughly a factor of two and should not be used for precise burden accounting.

### 4.6 Comparison With Other Settings and Biological Plausibility

The pooled prevalence falls within the 43%–79% band reported by individual national surveys (Sumon et al. 2021; Ali et al. 2023), and typical raw‐milk means of 35–120 ng L^−1^ are comparable to international compilations (Iqbal et al. 2015; Becker‐Algeri et al. 2016). The distinctive Bangladeshi feature is not prevalence, but the upper tail: the Dhaka mean near 699 ng L^−1^ (Tarannum et al. 2022) is extreme by any international standard, and the reconstruction of Section 3.5 indicates that it requires a ration‐weighted AFB_1_ of about 89 µg kg^−1^, attainable in the surveyed feed literature, but only at its upper extreme, which is itself a reason to treat that dataset as influential and to prioritise its independent replication. Because AFM_1_ acts multiplicatively with chronic hepatitis‐B infection (Liu and Wu 2010; EFSA Panel on Contaminants in the Food Chain [CONTAM] 2020) and Bangladesh carries an intermediate HBsAg prevalence, the population burden may exceed these point estimates; conversely, the HCC estimates inherit the full uncertainty of the potency factor and should be treated as order‐of‐magnitude indicators rather than as case counts.

### 4.7 Implications and Research Priorities

The findings support upstream control as the primary intervention: drying maize and ingredients to safe moisture, improved storage, mycotoxin binders and monsoon‐timed surveillance would curtail AFB_1_ before it reaches the cow (Gruber‐Dorninger et al. 2019), and the mechanistic reconstruction indicates that a ration‐weighted AFB_1_ below roughly 10 µg kg^−1^ would place predicted milk residues near or below the EU ML. The BSTI/BFSA milk standard (Bangladesh Food Safety Authority 2017) should be enforced and progressively tightened towards the EU level (European Commission 2006; FAO/WHO Joint Expert Committee on Food Additives [JECFA] 2018), with explicit protection for products intended for infants and young children, whose margins of exposure are an order of magnitude below those of adults.

The research priority follows directly from the design limitation identified in Section 4.2. What is required is not another single‐compartment survey but paired, probability‐based feed‐and‐milk sampling: AFB_1_ in the ration and AFM_1_ in the bulk milk of the same herds, repeated across the monsoon and dry seasons, with chromatographic confirmation, reported limits of detection and recovery, and sample‐level distributions rather than means alone. Such a design would replace the transferred carry‐over coefficient with a locally estimated one, allow the feed‐to‐milk relationship to be estimated rather than assumed, and convert the present screening‐level assessment into a quantitative national exposure model. Individual‐level consumption data for infants and young children, and chromatographic re‐analysis of the high‐concentration Dhaka milk‐shed, are the two highest‐value additions.

## 5 Strengths and Limitations

The principal strengths are the explicitly national focus, the formal quantification and structural explanation of heterogeneity, the use of Bangladesh‐specific consumption and body‐weight parameters, the combination of deterministic and probabilistic exposure modelling, and a structured uncertainty analysis that states the direction and magnitude of each source and tests whether any could reverse the conclusion (Table 4).

The limitations follow from the design and are set out in Sections 4.2 and 4.3. The assessment is literature‐based and modular, so no empirical feed‐to‐milk association is established. The evidence base is modest (20 clustered datasets, 605 samples), regionally uneven and entirely ELISA‐based, and includes six datasets at high risk of bias. Concentration data were available only as dataset means, so within‐dataset variability is not represented, and milk‐intake rates are representative rather than surveyed at individual level. The carry‐over model, the AFM_1_ relative potency factor and the HBsAg weighting are transferred assumptions whose uncertainty cannot be bounded from Bangladeshi data. The exposure estimate is consequently uncertain by roughly a factor of two, and the HCC estimates by considerably more; the finding that infant and child margins of exposure fall far below the safety benchmark is nonetheless robust to every sensitivity analysis performed.

## 6 Conclusions

Aflatoxin M_1_ was detected in the majority of Bangladeshi milk samples analysed to date (pooled prevalence 58.7%, 95% CI 51.6%–65.5%), although the wide prediction interval and the significant regional heterogeneity indicate that this figure summarises a heterogeneous literature rather than a uniform national condition. Eighty‐three per cent of sampled milk exceeded the EU ML, and the reported feed AFB_1_ burden is quantitatively consistent with the observed milk residues under published carry‐over coefficients, supporting the mechanistic plausibility of the pathway without establishing it empirically in Bangladeshi herds. Age‐stratified modelling places margins of exposure for infants and young children between 16‐ and 330‐fold below the MOE = 10,000 benchmark under every scenario, restriction, correction and probabilistic percentile examined, with the deterministic HI above unity in every age group and the probabilistic median above unity in every group except adults. On this screening‐level evidence, monsoon‐aware feed management, enforcement and progressive tightening of the national milk standard, and protection of young children are warranted now, while paired feed–milk surveillance with chromatographic confirmation is required to convert this literature‐based assessment into a definitive national exposure estimate.

## Author Contributions

**MD. Al Fattah**: conceptualizations, methodology, validation, writing – original draft, writing – review and editing, resources, data curation, formal analysis, visualization, software and investigation. **Md. Ahsan Habib**: conceptualizations, methodology, supervision, validation, funding acquisition, writing – original draft, project administration, writing – review and editing, software, resources, data curation, formal analysis and investigation.

## Funding

The authors have nothing to report.

## Ethics Statement

The authors have nothing to report.

## Conflicts of Interest

The authors declare no conflicts of interest.

## Supporting information




**Supporting File 1**: vms371284‐sup‐0001‐SuppMat.docx

**Supporting File 2**: vms371284‐sup‐0002‐SuppMat.docx

## Data Availability

The data that support the findings of this study are available on request from the corresponding author. The data are not publicly available due to privacy or ethical restrictions.
