# Supplementary material for: Aflatoxin M1 in Milk and Aflatoxin B1 in Animal Feed in Bangladesh: A Systematic Review, Meta‐Analysis and Literature‐Based Dietary Risk Assessment
Source: Vet Med Sci. 2026 Oct 16;12(6):e71284. doi: 10.1002/vms3.71284 (PMC13674741; doi:10.1002/vms3.71284)
Supplement: Supplementary file 1 [file VMS3-12-e71284-s001.docx]

**Supplementary Materials**

**Aflatoxin M₁ in Milk and Aflatoxin B₁ in Animal Feed in Bangladesh: A Systematic Review, Meta-Analysis and Literature-Based Dietary Risk Assessment**

*All estimates in Tables S2, S3 and S7 were computed from the dataset in Table 1 using the analysis pipeline described in Methods 2.3, and reproduce the corresponding figures in the main text. Tables S1, S4 and S6 reproduce values as reported in the primary sources or as executed in the search*

**Table S1.** Study-level extraction for the 20 AFM₁-in-milk datasets. Sample size, prevalence and mean concentration are as reported in the primary sources.

| ID | Division / area | Source | Milk type | n | Prevalence (%) | Mean AFM₁ (ng L⁻¹) | Risk of bias |
| --- | --- | --- | --- | --- | --- | --- | --- |
| 1 | Sylhet | Sumon et al. [15] | Raw | 26 | 72.0 | 53.7 | Low |
| 2 | Dhaka | Sumon et al. [15] | Raw | 28 | 71.0 | 37.8 | Low |
| 3 | Rajshahi | Sumon et al. [15] | Raw | 26 | 74.0 | 34.8 | Low |
| 4 | Chattogram | Sumon et al. [15] | Raw | 25 | 45.0 | 6.6 | Low |
| 5 | National | Sumon et al. [15] | Pasteurised | 15 | 100.0 | 106 | Low |
| 6 | National | Sumon et al. [15] | UHT | 15 | 100.0 | 73 | Low |
| 7 | Dhaka | Tarannum et al. [17] | Raw | 50 | 70.0 | 699.1 | Low |
| 8 | Dhaka | Tarannum et al. [17] | Pasteurised | 25 | 52.0 | 99.8 | Low |
| 9 | Dhaka | Tarannum et al. [17] | UHT | 25 | 20.0 | 35.5 | Low |
| 10 | Dhaka | Rahman et al. [16] | Raw | 50 | 64.0 | 152 | Moderate |
| 11 | Dhaka | Rahman et al. [16] | Pasteurised | 25 | 48.0 | 88 | Moderate |
| 12 | Chattogram | Ali et al. [18] | Raw | 30 | 47.0 | 190 | Moderate |
| 13 | Chattogram | Ali et al. [18] | Raw | 30 | 40.0 | 108.4 | Moderate |
| 14 | Chattogram | Ali et al. [18] | Raw | 30 | 43.0 | 189.3 | Moderate |
| 15 | Pabna–Sirajganj | Milk-shed survey | Raw | 45 | 75.0 | 120 | High |
| 16 | Mymensingh | Dairy-belt survey | Raw | 40 | 68.0 | 88 | High |
| 17 | Bogura | Regional survey | Raw | 35 | 66.0 | 95 | High |
| 18 | Khulna | Regional survey | Raw | 30 | 58.0 | 64 | High |
| 19 | Barishal | Regional survey | Raw | 25 | 50.0 | 58 | High |
| 20 | Rangpur | Regional survey | Raw | 30 | 55.0 | 70 | High |

*All 20 datasets used competitive ELISA and none reported chromatographic confirmation. Limits of detection, recovery and the treatment of left-censored values were reported too inconsistently across the primary reports to be tabulated uniformly and are therefore not included; this inconsistency is itself reported as a limitation (Results 3.1, Discussion 4.3). Risk of bias: Low = fully referenced peer-reviewed report with described sampling frame and method; Moderate = peer-reviewed report with incomplete methodological reporting; High = survey not attached to a fully referenced peer-reviewed report.*

**Table S1b.** Prevalence-adapted Newcastle–Ottawa appraisal: domains assessed and the basis for judgement.

| Appraisal domain | What was assessed | Basis for judgement across the 20 datasets |
| --- | --- | --- |
| Representativeness of the sample | Whether the sampling frame is described and relates to the milk supply of the stated area. | Described in the 14 datasets drawn from peer-reviewed reports; not described in the 6 survey-derived datasets. |
| Adequacy of sample size | Whether the sample size is justified against a stated precision target. | Not justified in any dataset; no report states a target precision. |
| Description of non-respondents / missing data | Whether excluded or unusable samples are accounted for. | Described only in the 9 datasets from Sumon et al. [15] and Tarannum et al. [17]. |
| Ascertainment of the outcome | Analytical method, its validation, and reporting of limits of detection and recovery. | All 20 datasets used competitive ELISA; none reported chromatographic confirmation, and limits of detection and recovery were reported too inconsistently to tabulate. |
| Completeness of outcome reporting | Whether sample size, prevalence and mean concentration are all recoverable. | Complete in the 14 peer-reviewed datasets; partial in the 6 survey-derived datasets, which report no dispersion. |

*The adapted instrument omits the comparability domain, which does not apply to single-group prevalence studies.*

**Table S1c.** Resulting risk-of-bias classification.

| Class | Datasets | IDs | Definition |
| --- | --- | --- | --- |
| Low | 9 | 1–9 | Fully referenced peer-reviewed report with described sampling frame and method |
| Moderate | 5 | 10–14 | Peer-reviewed report with incomplete methodological reporting |
| High | 6 | 15–20 | Survey not attached to a fully referenced peer-reviewed report |

*Excluding the six high-risk datasets leaves the pooled prevalence essentially unchanged but raises the sample-weighted mean concentration to 172 ng L⁻¹ (Results 3.8).*

**Table S2.** Division-level random-effects estimates. Prediction intervals are reported only where three or more datasets were available, consistent with Methods 2.3.

| Division / area | k | N | Prevalence % (95% CI) | 95% prediction interval | τ² | I² (%) | Q (df), p | Wtd. mean conc. (ng L⁻¹) |
| --- | --- | --- | --- | --- | --- | --- | --- | --- |
| Dhaka | 6 | 203 | 55.3 (41.0–68.9) | 15.7–89.2 | 0.378 | 73.1 | 18.56 (5), 0.002 | 242.3 |
| Chattogram | 4 | 115 | 43.7 (34.9–52.9) | 25.7–63.6 | 0.000 | 0.0 | 0.32 (3), 0.956 | 128.7 |
| National | 2 | 30 | 96.9 (79.9–99.6) | not estimable (k < 3) | 0.000 | 0.0 | 0.00 (1), 1.000 | 89.5 |
| Sylhet | 1 | 26 | 72.0 (52.2–85.8) | not estimable (k < 3) | 0.000 | 0.0 | n/a | 53.7 |
| Rajshahi | 1 | 26 | 74.0 (54.2–87.2) | not estimable (k < 3) | 0.000 | 0.0 | n/a | 34.8 |
| Pabna–Sirajganj | 1 | 45 | 75.0 (60.4–85.5) | not estimable (k < 3) | 0.000 | 0.0 | n/a | 120.0 |
| Mymensingh | 1 | 40 | 68.0 (52.2–80.5) | not estimable (k < 3) | 0.000 | 0.0 | n/a | 88.0 |
| Bogura | 1 | 35 | 66.0 (49.1–79.6) | not estimable (k < 3) | 0.000 | 0.0 | n/a | 95.0 |
| Khulna | 1 | 30 | 58.0 (40.1–74.0) | not estimable (k < 3) | 0.000 | 0.0 | n/a | 64.0 |
| Barishal | 1 | 25 | 50.0 (31.3–68.7) | not estimable (k < 3) | 0.000 | 0.0 | n/a | 58.0 |
| Rangpur | 1 | 30 | 55.0 (37.3–71.5) | not estimable (k < 3) | 0.000 | 0.0 | n/a | 70.0 |
| All datasets (overall) | **20** | **605** | **58.7 (51.6–65.5)** | **32.1–81.1** | **0.253** | **61.9** | **49.90 (19), <0.001** | **143.3** |

*Ten divisions or areas are represented, together with national samples, giving eleven strata. Single-dataset strata are shown for completeness; their intervals are exact binomial-equivalent logit intervals and carry no between-dataset variance component. The Dhaka and Chattogram estimates reproduce those given in Results 3.2.*

**Table S3.** Leave-one-out re-estimation of the pooled prevalence and of the sample-weighted mean concentration.

| Omitted | Division / area | Source | Milk type | Pooled prevalence % (95% CI) | I² (%) | τ² | Shift (pp) | Wtd. mean conc. (ng L⁻¹) |
| --- | --- | --- | --- | --- | --- | --- | --- | --- |
| 1 | Sylhet | Sumon et al. [15] | Raw | 58.0 (50.6–65.0) | 62.5 | 0.257 | −0.73 | 147.3 |
| 2 | Dhaka | Sumon et al. [15] | Raw | 58.0 (50.6–65.1) | 62.6 | 0.259 | −0.70 | 148.4 |
| 3 | Rajshahi | Sumon et al. [15] | Raw | 57.9 (50.6–64.9) | 62.0 | 0.252 | −0.85 | 148.2 |
| 4 | Chattogram | Sumon et al. [15] | Raw | 59.5 (52.1–66.4) | 62.4 | 0.258 | +0.75 | 149.2 |
| 5 | National | Sumon et al. [15] | Pasteurised | 58.1 (51.1–64.7) | 60.5 | 0.227 | −0.67 | 144.2 |
| 6 | National | Sumon et al. [15] | UHT | 58.1 (51.1–64.7) | 60.5 | 0.227 | −0.67 | 145.1 |
| 7 | Dhaka | Tarannum et al. [17] | Raw | 58.0 (50.5–65.1) | 61.8 | 0.261 | −0.78 | 93.2 |
| 8 | Dhaka | Tarannum et al. [17] | Pasteurised | 59.1 (51.7–66.2) | 63.6 | 0.271 | +0.40 | 145.2 |
| 9 | Dhaka | Tarannum et al. [17] | UHT | 60.4 (54.0–66.4) | 51.8 | 0.164 | +1.66 | 147.9 |
| 10 | Dhaka | Rahman et al. [16] | Raw | 58.4 (50.8–65.7) | 63.5 | 0.282 | −0.31 | 142.5 |
| 11 | Dhaka | Rahman et al. [16] | Pasteurised | 59.3 (51.9–66.4) | 63.0 | 0.265 | +0.60 | 145.7 |
| 12 | Chattogram | Ali et al. [18] | Raw | 59.4 (52.0–66.4) | 62.6 | 0.263 | +0.70 | 140.9 |
| 13 | Chattogram | Ali et al. [18] | Raw | 59.8 (52.6–66.6) | 60.4 | 0.239 | +1.05 | 145.1 |
| 14 | Chattogram | Ali et al. [18] | Raw | 59.6 (52.4–66.5) | 61.5 | 0.250 | +0.90 | 140.9 |
| 15 | Pabna–Sirajganj | Milk-shed survey | Raw | 57.6 (50.3–64.5) | 59.9 | 0.237 | −1.16 | 145.2 |
| 16 | Mymensingh | Dairy-belt survey | Raw | 58.2 (50.6–65.3) | 62.8 | 0.268 | −0.58 | 147.2 |
| 17 | Bogura | Regional survey | Raw | 58.3 (50.8–65.5) | 63.3 | 0.272 | −0.42 | 146.3 |
| 18 | Khulna | Regional survey | Raw | 58.8 (51.3–66.0) | 63.9 | 0.278 | +0.09 | 147.4 |
| 19 | Barishal | Regional survey | Raw | 59.2 (51.8–66.3) | 63.3 | 0.268 | +0.50 | 147.0 |
| 20 | Rangpur | Regional survey | Raw | 59.0 (51.4–66.2) | 63.8 | 0.277 | +0.26 | 147.1 |
| — | **None (all 20 datasets)** | **—** | **—** | **58.7 (51.6–65.5)** | **61.9** | **0.253** | **0.00** | **143.3** |

*Shift is the change in pooled prevalence in percentage points relative to the all-datasets estimate. The pooled prevalence remains within 57.6–60.4% throughout, whereas the weighted mean concentration ranges from 93 to 149 ng L⁻¹, the lower bound arising when dataset 7 (Dhaka raw milk, 699.1 ng L⁻¹) is omitted. This asymmetry is the basis for the statement in Results 3.8 that the exposure estimate is less stable than the prevalence estimate.*

**Table S4.** Extracted Bangladeshi feed mycotoxin dataset, reported descriptively and not pooled (Section 3.4).

| Feed / ingredient | Source | AFB₁ range (µg kg⁻¹) | Mean (µg kg⁻¹) | Exceeds 20 µg kg⁻¹ ML | Exceeds 5 µg kg⁻¹ (EU dairy) | Co-occurring toxins reported |
| --- | --- | --- | --- | --- | --- | --- |
| Poultry feed | [10,25] | 0–160 | ≈38 | Yes (mean and upper range) | Yes | Not reported |
| Maize | [8] | 1.07–103 | ≈28 | Yes (mean and upper range) | Yes | Fumonisins (to 9.18 mg kg⁻¹), zearalenone |
| Compound cattle feed | [11] | 7.78–14.66 | ≈11 | No | Yes | Not reported |
| Oil cakes / bran | [11] | 9.0–16 | ≈12 | No | Yes | Not reported |
| Rice straw (fodder) | [12] | ≈12.05 | ≈12 | No | Yes | Sterigmatocystin, zearalenone |
| Soybean meal | [11] | 4–15 | ≈9.8 | No | Yes (mean) | Not reported |

*These surveys differ in matrix definition, sampling frame and analytical method, and most do not report sample-level distributions, so they were not meta-analysed. Values are as given in the cited reports; ≈ denotes a reported approximate mean.*

**Table S5.** PRISMA 2020 checklist.

| Item | Checklist topic | Reported item | Location |
| --- | --- | --- | --- |
| 1 | Title | Identified as a systematic review, meta-analysis and risk assessment. | Title |
| 2 | Abstract | Structured summary with objectives, methods, pooled estimate, prediction interval and conclusions. | Abstract |
| 3 | Rationale | Fragmentation of the feed and milk literatures; absence of a national synthesis. | Introduction |
| 4 | Objectives | Four numbered objectives, with the modular design stated explicitly. | Introduction (final two paragraphs) |
| 5 | Eligibility criteria | Prevalence and/or concentration of AFM₁ in Bangladeshi milk, or AFB₁ in Bangladeshi feed, with extractable sample size; human breast milk excluded. | Methods 2.2 |
| 6 | Information sources | PubMed, Scopus, Web of Science Core Collection, ScienceDirect; searched 1 Jan 2000 – 31 Jan 2026; executed 31 Jan 2026; reference lists hand-searched; no registers. | Methods 2.2 |
| 7 | Search strategy | Full database-specific Boolean strings, filters and per-database record counts. | Table S6 |
| 8 | Selection process | Two reviewers, independent and in duplicate at title/abstract and full text; agreement reported; adjudication and conservative escalation rule described. | Methods 2.2 |
| 9 | Data collection process | Extraction by one reviewer, checked in full against source by the second. | Methods 2.2 |
| 10 | Data items | Division, source, milk type, n, prevalence, mean concentration, assay, risk-of-bias domains. | Table 1; Table S1 |
| 11 | Study risk of bias | Prevalence-adapted Newcastle–Ottawa instrument; three classes; domain-level scoring. | Methods 2.2; Table S1 |
| 12 | Effect measures | Prevalence, logit-transformed, with variance 1/[n p(1−p)]. | Methods 2.3, Equation 1 |
| 13 | Synthesis methods | DerSimonian–Laird random effects; back-transformation; prediction intervals; pre-specified subgroups; exploratory meta-regression; unit-of-analysis sensitivity analyses. | Methods 2.3 |
| 14 | Reporting bias assessment | Egger regression, Begg rank correlation, trim-and-fill, funnel inspection; limited power at k = 20 acknowledged. | Methods 2.7; Results 3.8 |
| 15 | Certainty assessment | Structured uncertainty tabulation following EFSA guidance. | Methods 2.7; Table 7 |
| 16 | Study selection | PRISMA flow: 318 identified, 96 duplicates, 222 screened, 34 sought, 32 assessed, 12 eligible reports, 20 datasets. | Figure 2; Methods 2.2 |
| 17 | Study characteristics | All 20 datasets characterised. | Table 1; Table S1 |
| 18 | Risk of bias in studies | Classified low / moderate / high; subgroup and restriction analyses reported. | Table 1; Results 3.2, 3.8 |
| 19 | Results of individual studies | Dataset-level prevalence and mean concentration. | Table 1; Figure 3 |
| 20 | Results of syntheses | Pooled prevalence 58.7% (95% CI 51.6–65.5); PI 32.1–81.1; I² 61.9%; τ² 0.253; subgroup and meta-regression results. | Results 3.2; Table 2; Table S2 |
| 21 | Reporting biases | Non-significant Egger and Begg; interpreted as non-probative. | Results 3.8; Figure 8 |
| 22 | Certainty of evidence | Uncertainty by nature, direction, magnitude and conclusion-reversing potential. | Table 7; Discussion 4.5 |
| 23 | Discussion | Principal findings, inferential limits, limitations of the primary literature, heterogeneity, uncertainty. | Discussion 4.1–4.5; Section 5 |
| 24 | Registration and protocol | The review was not prospectively registered; stated explicitly. | Methods 2.2 |
| 25 | Support | Funding statement. | Declarations |
| 26 | Competing interests | Declared. | Declarations |
| 27 | Availability of data and code | Extracted datasets, Boolean strings and analysis code. | Data availability |

**Table S6.** Full database-specific search strings, limits and yield.

| Database | Search string as executed | Field / limits | Date executed |
| --- | --- | --- | --- |
| PubMed | ("aflatoxin M1"[tiab] OR AFM1[tiab] OR "aflatoxin B1"[tiab] OR AFB1[tiab] OR mycotoxin*[tiab] OR "Aflatoxins"[Mesh] OR "Mycotoxins"[Mesh]) AND (milk[tiab] OR dairy[tiab] OR feed*[tiab] OR fodder[tiab] OR "Milk"[Mesh] OR "Animal Feed"[Mesh]) AND (Bangladesh[tiab] OR "Bangladesh"[Mesh]) AND ("2000/01/01"[Date - Publication] : "2026/01/31"[Date - Publication]) | Title/abstract + MeSH; publication date filter | 31 Jan 2026 |
| Scopus | TITLE-ABS-KEY(("aflatoxin M1" OR AFM1 OR "aflatoxin B1" OR AFB1 OR mycotoxin*) AND (milk OR dairy OR feed* OR fodder) AND Bangladesh) AND PUBYEAR > 1999 AND PUBYEAR < 2027 | TITLE-ABS-KEY; PUBYEAR limits | 31 Jan 2026 |
| Web of Science (Core Collection) | TS=(("aflatoxin M1" OR AFM1 OR "aflatoxin B1" OR AFB1 OR mycotoxin*) AND (milk OR dairy OR feed* OR fodder) AND Bangladesh) | Topic (TS); timespan 2000–2026; all editions | 31 Jan 2026 |
| ScienceDirect (run 1) | ("aflatoxin M1" OR AFM1) AND (milk OR dairy) AND Bangladesh | All fields; years 2000–2026 | 31 Jan 2026 |
| ScienceDirect (run 2) | ("aflatoxin B1" OR AFB1 OR mycotoxin) AND (feed OR dairy) AND Bangladesh | All fields; years 2000–2026 | 31 Jan 2026 |
| Records identified across all databases and runs | 318 | — | — |
| Duplicates removed before screening | **96** | **—** | **—** |
| Unique records screened | **222** | **—** | **—** |

*ScienceDirect supports neither truncation nor more than eight Boolean connectors within a single query, so the concept string was executed there as two complementary runs whose outputs were combined before deduplication. Totals correspond to the identification and screening counts in Figure 2.*

**Table S7.** Feed-to-milk carry-over model (Equation 6): parameters, goodness of fit, calibration range and propagation.

| Model component | Value | Source and notes |
| --- | --- | --- |
| Functional form | CO (%) = a · exp(b · Y) | Y = daily milk yield (kg day⁻¹); CO = percentage of ingested AFB₁ recovered as AFM₁ in milk |
| Coefficient a | 0.5154 | As published in [23] |
| Coefficient b | 0.0521 per kg day⁻¹ | As published in [23] |
| Coefficient of determination | r² = 0.622 | As reported in [23]; the same authors found a linear specification to fit less well |
| Calibration data | 12 Israeli-Holstein cows | Mid- and late-lactation; ~86 µg AFB₁ daily for 7 days; carry-over evaluated at steady state, days 3–7 |
| Reported group means | 5.8% (mid-lactation); 2.5% (late-lactation) | Steady-state means reported in [23] |
| Implied calibration yield range | ≈ 30–46 kg day⁻¹ | Obtained by back-substituting the two reported group means into the fitted function; consistent with the early-lactation worked example of 45 kg day⁻¹ in [23] |
| Coefficient standard errors and residual diagnostics | Not available | Not reported in the source, and not recomputable here because the animal-level pairs of yield and carry-over are not published; the relationship is used as published rather than refitted |
| Yield range applied in this study | 5–15 kg day⁻¹ | Below the calibration range: an extrapolation, not an interpolation, and reported as such in Methods 2.4 |
| External plausibility of the extrapolation | 0.67–1.13% predicted at 5–15 kg day⁻¹ | Against a reviewed central estimate of 1–2% for dairy cows generally, rising to about 6% at high production [42]; the extrapolation therefore predicts less transfer than the pooled literature implies |
| Propagation into EDI / MOE / HI / HCC | None, by design | Exposure is computed from the milk module alone (Equation 8); the model enters only the consistency check, where an erroneous coefficient would appear as a failure of observed dataset means to fall within the predicted envelope |

*The carry-over function does not enter the exposure assessment, so an error in these coefficients would not alter the risk estimates. Its use at Bangladeshi smallholder yields is an extrapolation below the range over which it was calibrated; this is stated in Methods 2.4 and quantified below.*

**Table S7b. Carry-over predicted by Equation 6 across the milk-yield range.**

| Milk yield (kg day⁻¹) | Predicted carry-over (%) | Relation to calibration range | Context |
| --- | --- | --- | --- |
| 3 | 0.60 | Extrapolated below | Range typical of Bangladeshi smallholder cattle; used in Section 3.5 |
| 5 | 0.67 | Extrapolated below | Range typical of Bangladeshi smallholder cattle; used in Section 3.5 |
| 7 | 0.74 | Extrapolated below | Range typical of Bangladeshi smallholder cattle; used in Section 3.5 |
| 10 | 0.87 | Extrapolated below | Range typical of Bangladeshi smallholder cattle; used in Section 3.5 |
| 12 | 0.96 | Extrapolated below | Range typical of Bangladeshi smallholder cattle; used in Section 3.5 |
| 15 | 1.13 | Extrapolated below | Range typical of Bangladeshi smallholder cattle; used in Section 3.5 |
| 18 | 1.32 | Extrapolated below | Between smallholder and calibrated yields |
| 20 | 1.46 | Extrapolated below | Between smallholder and calibrated yields |
| 25 | 1.90 | Extrapolated below | Between smallholder and calibrated yields |
| 30 | 2.46 | Within | High-yielding dairy cattle, as in the calibration data |
| 35 | 3.19 | Within | High-yielding dairy cattle, as in the calibration data |
| 40 | 4.14 | Within | High-yielding dairy cattle, as in the calibration data |
| 45 | 5.37 | Within | High-yielding dairy cattle, as in the calibration data |
| 46.5 | 5.81 | Within | High-yielding dairy cattle, as in the calibration data |

*At the 5–15 kg day⁻¹ yields typical of Bangladeshi smallholder cattle the function returns 0.67–1.13%, the range quoted in Results 3.5. Rows at and above 30 kg day⁻¹ fall within the range over which the function was calibrated and are shown for comparison; the predicted 2.46% at 30 kg and 5.81% at 46.5 kg reproduce the two group means reported in [23].*

**Supplementary Figures**

**Figure S1.** Mean AFM₁ concentration by division and milk type across the 20 datasets; symbol area is proportional to sample size and the concentration axis is logarithmic. Descriptive counterpart to Table 1. The single Dhaka raw-milk dataset near 700 ng L⁻¹ is the only value above the BSTI/BFSA and Codex maximum level, and it is the dataset whose removal lowers the sample-weighted mean from 143 to 93 ng L⁻¹.

**Figure S2.** Reported AFB₁ in Bangladeshi feeds and feed ingredients on a logarithmic scale; bars span the reported range and dots mark the reported mean. Descriptive counterpart to Table 3. Poultry feed and maize breach the 20 µg kg⁻¹ general feed maximum level, while every matrix exceeds the stricter 5 µg kg⁻¹ EU limit for dairy-cattle feed. The arrow on the poultry-feed bar indicates a reported lower bound of zero, which cannot be shown on a logarithmic axis.

**Figure S3.** Deterministic age-stratified exposure and risk, descriptive counterpart to Table 4. (A) Estimated daily intake under the central (143 ng L⁻¹) and high (250 ng L⁻¹) concentration scenarios. (B) Corresponding margins of exposure on a logarithmic scale, with the MOE = 10,000 benchmark marked; no age group approaches it under either scenario.

Appendix B Estimator and unit-of-analysis sensitivity analyses

Pooled prevalence of AFM_1_ in Bangladeshi milk on the logit scale (*k* = 20 datasets, *N* = 605), under alternative between-study variance estimators and alternative units of analysis. Corresponds to results 3.8.

| Specification | *τ*² | Prevalence (95% CI) | 95% prediction interval |
| --- | --- | --- | --- |
| DerSimonian–Laird (primary) | 0.253 | 58.7% (51.6–65.5) | 32.1%–81.1% |
| REML | 0.227 | 58.7% (51.8–65.3) | 33.3%–80.2% |
| Paule–Mandel | 0.423 | 59.0% (50.5–67.0) | 25.9%–85.6% |
| REML + Hartung–Knapp | 0.227 | 58.7% (50.2–66.7) | 33.3%–80.2% |
| Paule–Mandel + Hartung–Knapp | 0.423 | 59.0% (49.9–67.6) | 25.9%–85.6% |
| Aggregated within source (*k* = 10) | 0.167 | 60.3% (52.6–67.5) | 35.6%–80.7% |
| Largest stratum per source (*k* = 10) | 0.043 | 63.3% (57.5–68.7) | — |
